# Supplementary material for: AGREEing on Clinical Practice Guidelines for Autism Spectrum Disorders in Children: A Systematic Review and Quality Assessment
Source: Children (Basel). 2022 Jul 14;9(7):1050. doi: 10.3390/children9071050 (PMC9323940; doi:10.3390/children9071050)
Supplement: Supplementary file 1 [file children-09-01050-s001.zip › children-1783030-supplementary.pdf]

Supplementary Table S1 Search Strategy

Keywords	autism, autism spectrum disorder, ASD, autistic disorder, infantile autism, childhood autism, pervasive developmental disorder (PDD), Asperger's syndrome, and PDD not otherwise specified (PDD-NOS), pediatrics, pediatric medicine, child health, mental health, treatment, management, pharmacology, practice guidelines, clinical practice guidelines, healthcare quality, patient safety, evidence-based medicine, AGREE II instrument, quality assessment, critical appraisal, evidence-based pediatrics, evidence-based psychiatry.
Search strategy (MEDLINE)	(((((((((((autism[Title/Abstract]) OR (autism spectrum disorder[Title/Abstract])) OR (ASD[Title/Abstract])) OR (autistic disorder[Title/Abstract])) OR (infantile autism[Title/Abstract])) OR (childhood autism[Title/Abstract])) OR (pervasive developmental disorder[Title/Abstract])) OR (PDD[Title/Abstract])) OR (Asperger's syndrome[Title/Abstract])) OR (PDD[Title/Abstract] NOT otherwise specified[Title/Abstract])) OR (PDD-NOS[Title/Abstract])) AND ((((pediatrics) OR (pediatric medicine)) OR (child health)) OR (mental health))) AND ((practice guidelines) OR (clinical practice guidelines) OR (evidence-based medicine)). MY NCBI Filters: *Article Type*: Practice Guideline. *Publication date*: From 1/11/2015 to the present date.

Results of the search: Total number of retrieved articles: 165, Total number of excluded articles: 161, and Total number of included CPGs: 4

Supplementary Table S2. Items of the PIPOH Model for eligible ASD CPGs

PIPOH Items	Decsription
**Population (P)**	Children and young people of both genders with ASD aged 2 to 18 years with or without the common comorbidities like Attention Deficit Hyperactivity Disorder (ADHD), anxiety, and other relevant medical problems.
**Intervention(s) (I)**	Early identification, diagnosis, differential diagnosis, investigations, pharmacological and non-pharmacological therapy treatment, sleep management, among others.
**Professionals (P) or Target users of CPGs, and Clinical specialties**	Child psychiatrists, behavioral developmental pediatricians, pediatric neurologists, medical geneticists, physical medicine and rehabilitation therapists, mental health specialists, clinical pharmacists, nurses, speech therapists, special educators, occupational therapists, patients, and families.
**Outcomes (O)**	Early identification and diagnosis, prevention of death, prevention major neurodevelopmental disabilities in surviving babies, and appropriateness of prescription of pharmacotherapy
**Healthcare context (H)**	Primary, secondary and tertiary healthcare settings.

Supplementary Table S3. Items of the PICAR statement for eligible ASD CPGs

PICAR Item	Decsription
**P: Population, clinical indication(s), and condition(s)**	**Study population:** Children and young people aged 2–18 years.<br>**Clinical indication:** Early identification, assessment, diagnosis, and management.<br>**Clinical condition:** ASD with or without the common comorbidities like ADHD, anxiety, and other relevant medical problems.
**I: Intervention(s)**	All interventions are of interest pharmacological and non-pharmacological.
**C: Comparator(s), comparison(s), and (key) content**	**The following Key clinical content is of interest in eligible CPGs: -**<br>• Early identification, assessment, and diagnosis<br>• Pharmacological interventions<br>• Non-pharmacological interventions<br>• Transition of care
**A: Attributes of the CPG**	**CPG eligibility (inclusion) criteria: -**<br>Evidence-based with a clear record of their development methods<br>Published in English or Arabic language.<br>Original source CPGs (de novo development).<br>National or international scope and purpose.

	Published by an organization or group authorship and accessible from a CPG database or peer-reviewed journal. Only the most current version of each source CPG was appraised.<br>**CPG exclusion criteria: -**<br>Published in or before October 2015.<br>Not in the English or Arabic languages.<br>Adapted from other CPGs.<br>Proposed as consensus or expert-based statements.<br>Single author CPG.
**R: Recommendation characteristics and**<br>**''other'' considerations**	CPG eligibility is dependent on the presence of eligible CPG Recommendations for early diagnosis, assessment, pharmacological and non-pharcaological interventions.

Abbreviations: CPGs, clinical practice guidelines; ADHD: Attention Deficit Hyperactivity Disorder, ASD: Autism Spectrum Disorder

Supplementary Table S4. Interpretation of the strength of agreement according to K value.

Value of K	Strength of agreement
< 0.20	Poor
0.21 - 0.30	Fair
0.31 - 0.40	Moderate
0.41 - 0.60	Good
0.61 - 0.80	Very good
0.81 – 1.00	Excellent

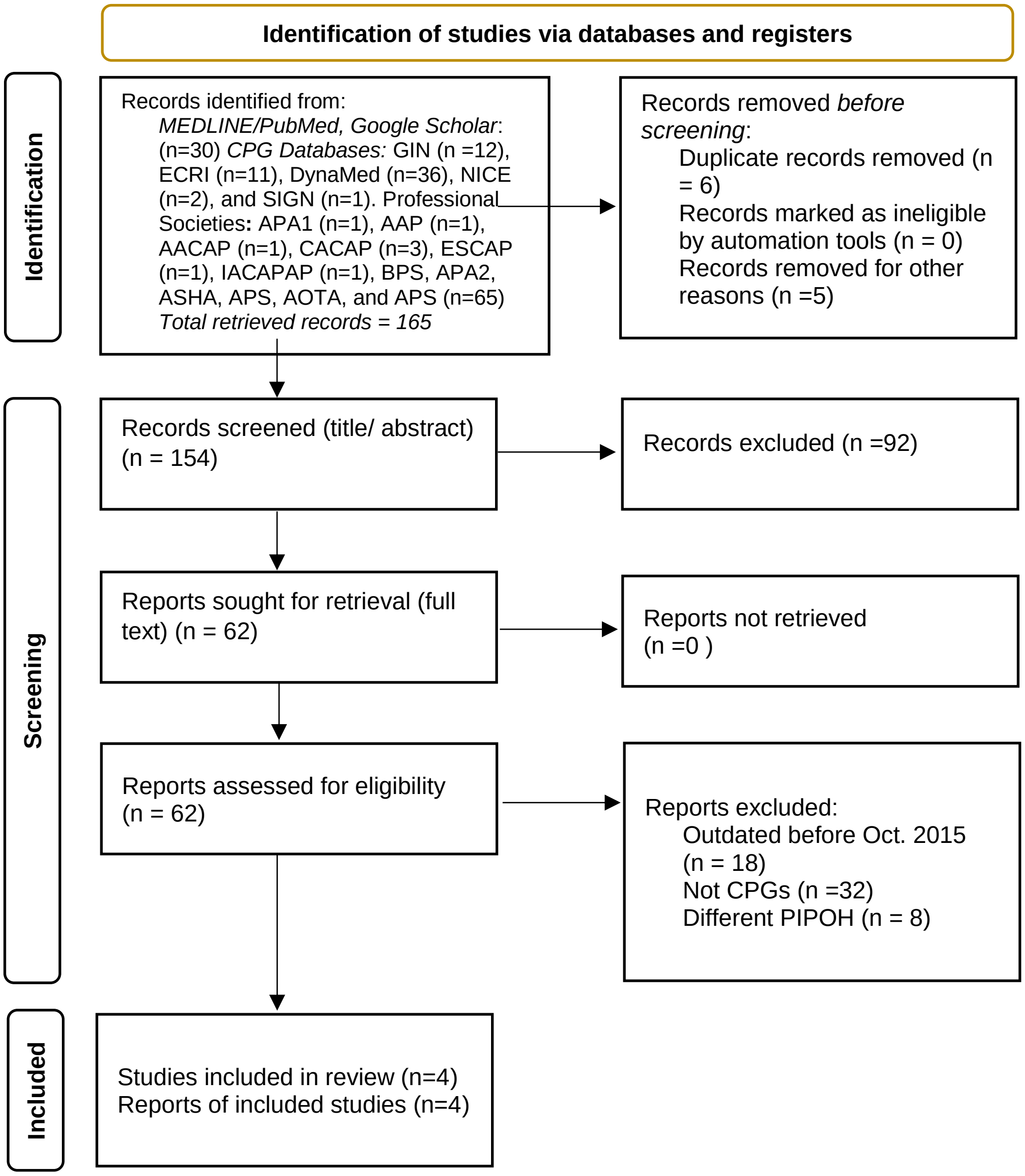

Supplementary Figure S1. PRISMA 2020 flow diagram for new systematic reviews which included searches of databases and registers only

From: Page MJ, McKenzie JE, Bossuyt PM, Boutron I, Hoffmann TC, Mulrow CD, et al. The PRISMA 2020 statement: an updated guideline for reporting systematic reviews. BMJ 2021;372:n71. doi: 10.1136/bmj.n71

For more information, visit: http://www.prisma-statement.org/

Supplementary Table S5. Recommendation matrix comparing the four appraised CPGs: ACRC, NICE, NZMOH, and SIGN

	NICE	SIGN-HIS	NZ MOH	ACRC
**EARLY IDENTIFICATION & DIAGNOSIS**				
**Early recognition and diagnosis**	- A local autism multi-agency strategy group should be set up, with managerial, commissioner and clinical representation from child health and mental health services, education, social care, parent and career service users, and the voluntary sector.<br>- The local autism strategy group should appoint a lead professional to be responsible for the local autism pathway for recognition, referral, and diagnosis of children and young people.<br>- The aims of the group should include:<br>- Improving early recognition of autism by raising awareness of the signs and symptoms of	- Population screening for ASD is not recommended.<br>- As part of the core program of child health surveillance, healthcare professionals can aid early identification of children requiring further assessment for ASD and other developmental disorders.<br>- Clinical assessment should incorporate a high level of vigilance for features suggestive of ASD, in the domains of social interaction and play, speech, language and communication difficulties and behavior.<br>- The assessment of children and young people with developmental delay, emotional and behavioral problems, psychiatric	- Early identification of children with autism spectrum disorder is essential.<br>- Early identification enables early intervention and is likely to lead to better function in later life.<br>- Early identification is achieved by:<br>a. comprehensive developmental surveillance of all children so deviations from normal development are recognized early b. valuing and addressing parental concerns about their child's development	- It is suggested that the Assessment Team use the current versions of either of the following international diagnostic manuals to make diagnostic decisions in relation to ASD:<br>a. find Statistical Manual of Mental Disorders.<br>b. International Statistical Classification of Diseases and Related Health Problems.<br>- It is recommended that the process for assessing ASD concerns follow an evidence-based approach, where clinical decision-making is based on a review of the best available research evidence.<br>- It is recommended that the process for assessing ASD concerns follow an individual- and family-centered approach, by

	NICE	SIGN-HIS	NZ MOH	ACRC
	autism through multi-agency training.<br>- Making sure the relevant professionals (healthcare, social care, education, and voluntary sector) are aware of the local autism pathway and how to access diagnostic services.<br>- Supporting the smooth transition to adult services for young people going through the diagnostic pathway<br>- Ensuring data collection and audit of the pathway takes place.<br>- In each area a multidisciplinary group (the autism team) should be set up. The core membership should include:<br>a. pediatrician and/or child and adolescent psychiatrist<br>b. speech and language therapist, clinical and/or educational psychologist.	disorders, impaired mental health or genetic syndromes should include surveillance for ASD as part of routine practice.<br>- Healthcare professionals should consider informing families that there is a substantial increased risk of ASD in siblings of affected children	c. prompt access to diagnostic services.	which assessment professionals collaborate with individuals and their families to identify the unique needs, strengths and contexts of the person undergoing assessment and their broader family unit.<br>- It is recommended that the process for assessing ASD concerns follow a holistic framework, where an individual is evaluated within their personal, activity and environmental contexts, and that referrals for further supports are based on an individual’s functioning and needs, rather than their clinical diagnosis.<br>- It is recommended that the process for assessing ASD concerns follow a strengths-focused approach, in which identifying the strengths, skills, interests, resources and support systems of the individual and their caregiver(s) and/or support

	NICE	SIGN-HIS	NZ MOH	ACRC
	- The autism team should either include or have regular access to the following professionals if they are not already in the team: pediatrician or pediatric neurologist‹ child and adolescent psychiatrist‹ educational psychologist, clinical psychologist, occupational therapist.<br>- Consider including in the autism team (or arranging access for the team to) other relevant professionals who may be able to contribute to the autism diagnostic assessment. For example, a specialist health visitor or nurse, specialist teacher, or social worker.<br>- The autism team should have the skills and competencies to:<br>a. carry out an autism diagnostic assessment			people is recognized as being as important as identifying limitations.<br>- It is recommended that the process for assessing ASD concerns be accessible and rigorous for all Australians regardless of age, gender, cultural background, socioeconomic status or geographical location.<br>- It is recommended that the process for assessing ASD concerns take a lifespan perspective, where consideration is given to the individual's present and future challenges and opportunities.<br>- It is recommended that the process for assessing ASD concerns incorporate:<br>a. Comprehensive Needs Assessment.<br>b. Diagnostic Evaluation.<br>- It is suggested that the process for assessing ASD concerns be coordinated by a nominated

	NICE	SIGN-HIS	NZ MOH	ACRC
	b. communicate with children and young people with suspected or known autism, and with their parents and careers, and sensitively share the diagnosis with them.<br>- Autism team members should:<br>a. provide advice to professionals about whether to refer children and young people for autism diagnostic assessments<br>b. decide on the assessment needs of those referred or when referral to another service will be needed<br>c. carry out the autism diagnostic assessment<br>d. share the outcome of the autism diagnostic assessment with parents and careers, and with children and young people if appropriate<br>e. with parent or career consent and, if appropriate,			clinician from the initial referral for an assessment until findings have been communicated to the individual and/or their caregiver(s).

	NICE	SIGN-HIS	NZ MOH	ACRC
	the consent of the child or young person, share information from the autism diagnostic assessment directly with relevant services, for example through a school visit by an autism team member.<br>f. offer information to children, young people and parents and careers about appropriate services and support.<br>- Provide a single point of referral for access to the autism team.<br>- The autism team should either have the skills (or have access to professionals that have the skills) needed to carry out an autism diagnostic assessment, for children and young people with special circumstances including:<br>a. coexisting conditions such as severe visual and hearing impairments, motor disorders			

	NICE	SIGN-HIS	NZ MOH	ACRC
	including cerebral palsy, severe learning (intellectual) disabilities, complex language disorders or complex mental health disorders<br>b. looked-after children and young people.<br><br>- When referring children and young people to the autism team, include in the referral letter the following information:<br>a. reported information from parents, careers and professionals about signs and/or symptoms of concern<br>b. your own observations of the signs and/or symptoms.<br>- When referring children and young people to the autism team, include in the referral letter the following information, if available:<br>a. antenatal and perinatal history			

	NICE	SIGN-HIS	NZ MOH	ACRC
	b. developmental milestones<br>c. factors associated with an increased prevalence of autism<br>d. relevant medical history and investigations<br>e. information from previous assessments.<br>- Explain to parents or careers and, if appropriate, the child or young person, what will happen on referral to the autism team or another service.<br>- If you do not think concerns are sufficient to prompt a referral, consider a period of watchful waiting. If you remain concerned about autism, reconsider your referral decision.<br>-If the parents or careers or if appropriate, the child or young person, prefer not to be referred to the autism team, consider a period of watchful			

	NICE	SIGN-HIS	NZ MOH	ACRC
	waiting. If you remain concerned about autism, reconsider referral.<br>- If a concern about possible autism has been raised but there are no signs, symptoms or other reasons to suspect autism, use professional judgment to decide what to do next.			
**ASSESSMENT, AND SCORING ALGORITHMS**				

	NICE	SIGN-HIS	NZ MOH	ACRC
**Use of multidisciplinary team assessment**	- When a child or young person is referred to the autism team, at least one member of the autism team should consider whether to carry out: an autism diagnostic assessment and/or an alternative assessment.<br>- Carry out an autism diagnostic assessment if there is regression in language or social skills in a child younger than 3 years.<br>- Refer first to a pediatrician or pediatric neurologist (if this has not already happened) children or young people: older than 3 years with regression in language of any age with regression in motor skills.<br>- The pediatrician or pediatric neurologist can refer back to the autism team if necessary.<br>- When deciding whether to carry out an autism diagnostic	- If, on the basis of initial assessment, it is suspected that the individual may have ASD, they should be referred for specialist assessment.<br>- A diagnostic assessment, alongside a profile of the individual's strengths and weaknesses, carried out by a multidisciplinary team which has the skills and experience to undertake the assessments, should be considered as the optimum approach for individuals suspected of having ASD.<br>- The use of different professional groups in the assessment process is recommended as it may identify different aspects of ASD and aid accurate diagnosis.<br>- Specialist assessment should involve a history-taking element, a clinical	- All children suspected of having ASD or another developmental problem should have an audiology assessment.<br>- Diagnostic assessment of young people and adults should be the person concerned in interview and observation.<br>- Preferably, a multidisciplinary team of health care practitioners experienced in ASD should undertake diagnostic assessment of young people and adults suspected of having ASD.<br>- Without an assessment team, a health care practitioner trained and highly experienced in ASD may undertake diagnostic assessment	- It is recommended that all clinicians involved in assessment of ASD concerns obtain relevant training and expertise covering all the following areas:<br>– typical and atypical development across the age range assessed in their practice<br>– presentation of the signs and/or symptoms of ASD and other neurodevelopmental disorders across all developmental stages in which they practice, along with the manifestations of these symptoms during early development (which is relevant to diagnostic criteria).<br>– presentation of symptoms of ASD and other neurodevelopmental disorders among male, female and, where applicable, gender-diverse individuals<br>– the impact of other important considerations, such as intellectual and/or

	NICE	SIGN-HIS	NZ MOH	ACRC
	assessment, take account of the following (unless the child is under 3 years and has regression in language or social skills:<br>a. the severity and duration of the signs and/or symptoms the extent to which the signs and/or symptoms are present across different settings<br>b. the impact of the signs and/or symptoms on the child or young person and on their family or career<br>- Start the autism diagnostic assessment within 3 months of the referral to the autism team.<br>- A case coordinator in the autism team should be identified for every child or young person who is to have an autism diagnostic assessment.<br>- The autism case coordinator should:	observation/assessment element, and the obtaining of wider contextual and functional information.<br>- Specialist assessment should be available for any individuals who need it.<br>Specialist teams should assess if their service is being used equitably.<br>- Apparent inequalities should be investigated and addressed.<br>- An assessment of mental health needs, well-being and risk should be considered for all individuals with ASD presenting to any agency.<br>- Healthcare professionals involved in specialist assessment should take an ASD-specific developmental history and should directly observe and assess the individual's social and communication skills and behavior.	- All District Health Boards (DHBs) should have in place processes that ensure:<br>a. referral pathways for children and adults who may have ASD or another developmental problem are clearly understood by professionals<br>b. services are coordinated within and across sectors<br>c. multidisciplinary, multiagency assessments are provided<br>d. all services are provided in a timely manner.	communication capacity, culturally, linguistically and/or socio-economically diverse background, regional or remote location, or complex psychosocial factors, on the assessment of ASD concerns<br>- assessment of ASD and other neurodevelopmental disorders<br>- administration of standardized assessments (with all prerequisites for using the instrument in clinical practice met)<br>- clinical reasoning in weighing evidence, integrating findings and reaching assessment conclusions<br>- clinical report writing<br>-communicating with individuals on the autism spectrum and their caregivers.<br>- It is recommended that all clinicians involved in assessment of ASD concerns, in addition to the foundation qualification(s) relevant to their professional

	NICE	SIGN-HIS	NZ MOH	ACRC
	a. act as a single point of contact for the parents or careers and, if appropriate, the child or young person being assessed, through whom they can communicate with the rest of the autism team<br>b. keep parents or careers and, if appropriate, the child or young person, up-to-date about the likely time and sequence of assessments<br>c. arrange the provision of information and support for parents, careers, children and young people as directed by the autism team<br>d. gather information relevant to the autism diagnostic assessment<br>- Discuss with the parents or careers and, if appropriate, the child or young person, how information should be shared throughout the autism diagnostic assessment,	- Consider the use of a structured instrument to assist information gathering in the assessment of an individual with possible ASD.<br>- Information about individual's functioning outside the clinic setting, should routinely be obtained from as many available sources as is feasible.		discipline, obtain and maintain relevant training and expertise through peer observation, peer supervision and peer mentoring. Formal training courses and/or further qualifications may supplement these peer learning approaches.<br>- It is suggested that the clinician who conducted the Single Clinician Diagnostic Evaluation invite additional clinician(s) as required to participate in the Consensus Team Diagnostic Evaluation, based on the match between professional expertise and the area(s) of diagnostic uncertainty identified during the Single Clinician Diagnostic Evaluation. This should involve at least one other professional from a different discipline or specialty to the clinician who conducted the Single Clinician Diagnostic Evaluation.

	NICE	SIGN-HIS	NZ MOH	ACRC
	including communicating the outcome of the assessment. Take into account, for example, the child or young person's age and ability to understand.			- It is recommended that a Consensus Team Diagnostic Evaluation include at least one additional clinician who meets at least one of the following eligibility criteria:<br>a. medical practitioner who holds specialist registration with the Medical Board of Australia in the field of community child health, general pediatrics, psychiatry or neurology<br>b. medical practitioner who holds general or specialist registration with the Medical Board of Australia and has at least six years of relevant experience, training or supervision in the assessment of neurodevelopmental disorders<br>c. occupational therapist who holds registration with the Occupational Therapy Board of Australia<br>d. psychologist who holds general registration, with or without a

	NICE	SIGN-HIS	NZ MOH	ACRC
				practice endorsement, with the Psychology Board of Australia<br>e. speech pathologist who is eligible to be a Certified Practicing Member of Speech Pathology Australia.<br>- It is recommended that a Consensus Team Diagnostic Evaluation be conducted by clinicians who, in addition to the relevant training and expertise required by all members of the Assessment Team, have relevant training and expertise in all the following areas:<br>a. clinical reasoning in weighing evidence, performing diagnostic formulations and making diagnostic decisions<br>b. signs and symptoms associated with common co-occurring or differential diagnosis conditions<br>c. the criteria for ASD and co-occurring or differential diagnosis conditions described by the current version of international

	NICE	SIGN-HIS	NZ MOH	ACRC
				diagnostic manuals (e.g. DSM and/or ICD).<br>- It is recommended that all clinicians involved in assessment of ASD concerns, in addition to the foundation qualification(s) relevant to their professional discipline, obtain and maintain relevant training and expertise through peer observation, peer supervision and peer mentoring. Formal training courses and/or further qualifications may supplement these peer learning approaches.<br>- It is recommended that members of the Consensus Diagnosis Team obtain and maintain the additional skills and expertise listed in Recommendation 49 through peer observation, peer supervision and peer mentoring. Formal training courses and/or further qualifications may

	NICE	SIGN-HIS	NZ MOH	ACRC
				supplement these peer learning approaches.<br>- Multidisciplinary team members:<br>a. Medical practitioner<br>b. Nurse<br>c. Occupational therapist<br>d. Psychologist<br>e. Social worker<br>f. Speech pathologist<br>- For nurses conducting a Comprehensive Needs Assessment, it is recommended that they be a nurse practitioner (with appropriate credentials in neurodevelopmental disorder assessment) or a clinical nurse specialist/consultant (practicing under appropriate medical supervision).<br>- To conduct a Diagnostic Evaluation, medical practitioners are recommended to have specialist registration in the field of community child health, general pediatrics, psychiatry or neurology, or have general

	NICE	SIGN-HIS	NZ MOH	ACRC
				registration with at least six years of relevant experience in the assessment of neurodevelopmental disorders.<br>- To conduct a Single Clinician Diagnostic Evaluation, psychologists are recommended to have a practice endorsement in clinical psychology, educational/developmental psychology or neuropsychology.<br>- The Assessment Team will liaise with other medical, allied health, disability and/or educational professionals to obtain further information about the individual being assessed, to support the Comprehensive Needs.<br>- These other professionals are not part of the Assessment Team; however, their input may be helpful to obtain a more complete clinical picture of the individual's presentation in their everyday environment or provide specialist guidance to explore alternative

	NICE	SIGN-HIS	NZ MOH	ACRC
				explanations for presenting signs and/or symptoms.
**Telehealth setting Regional and remote locations**	Not mentioned	Not mentioned	Not mentioned	- A telehealth setting refers to interactions using telephone conversations or video conferencing, and/or reviewing video recordings.<br>- It is suggested that telehealth may be used to complement face-to-face meetings, but is not to be used as the sole medium for conducting a Single Clinician Diagnostic Evaluation and/or Consensus Team Diagnostic Evaluation.<br>- It is important that at least one face-to-face assessment session is conducted with a Single Diagnostician and/or member of the Consensus Diagnosis Team.<br>- The use of telehealth as the predominant medium for conducting an assessment of ASD concerns should be restricted to

	NICE	SIGN-HIS	NZ MOH	ACRC
				exceptional circumstances, such as when conducting a face-to-face assessment in a clinic or community setting would be very difficult.<br>- Examples include when a client lives in a regional or remote location without access to assessment teams, or other significant travel restrictions prevent a face-to-face assessment occurring (such as challenges related to sensory or anxiety symptoms).<br>- If telehealth is used as the predominant medium for conducting part of an assessment of ASD concerns or sharing the findings, it is recommended that a local clinician (or other professional with relevant expertise) be physically present with the client during the telehealth meetings.<br>- It is important that clinicians and other professionals undertake

	NICE	SIGN-HIS	NZ MOH	ACRC
				appropriate training to ensure they have the appropriate knowledge and skills to implement the Guideline within their service:<br>a. this may involve tailoring the available resources to meet the needs of their local community and type of service.<br>b. this will help ensure ongoing capacity within all communities.<br>- It is important to develop in-person or online clinical networks to facilitate the training of new members of an assessment team and maintain required expertise and mentoring systems:<br>a. this is in recognition that peer-to-peer learning is critical to developing and maintaining high levels of clinical skills, in particular through peer observation, peer supervision and peer mentoring.
**Risk factors associated with ASD**	• A sibling with autism.<br>• Birth defects associated with central nervous system	• preterm infants, adverse events in utero or trauma at birth	Not mentioned	Not mentioned

	NICE	SIGN-HIS	NZ MOH	ACRC
	malformation and/or dysfunction, including cerebral palsy.<br>• Gestational age less than 35 weeks.<br>• Parental schizophrenia-like psychosis or affective disorder.<br>• Maternal use of sodium valproate in pregnancy.<br>• A learning (intellectual) disability.<br>• Attention deficit hyperactivity disorder.<br>• Neonatal encephalopathy or epileptic encephalopathy, including infantile spasms.<br>• Chromosomal disorders such as Down's syndrome.<br>• Genetic disorders such as fragile X.<br>• Muscular dystrophy.<br>• Neurofibromatosis.<br>• Tuberous sclerosis.	• familial history of autism/ASD<br>• children whose parents have mental health problems<br>• maternal use of antidepressants or anticonvulsants		

	NICE	SIGN-HIS	NZ MOH	ACRC
**Referral for diagnostic clarification**	- If any of the following apply after assessment, consider obtaining a second opinion (including referral to a specialized tertiary autism team if necessary):<br>a. continued uncertainty about the diagnosis<br>b. disagreement about the diagnosis within the autism team<br>c. disagreement with parents or careers or, if appropriate, the child or young person, about the diagnosis<br>d. a lack of local access to particular skills and competencies needed to reach a diagnosis in a child or young person who has a complex coexisting condition, such as a severe sensory or motor impairment or mental health problem<br>e. a lack of response as expected to any therapeutic	- Children under three years of age who have regression in language or social skills should be referred for assessment for ASD.<br>- Instruments may be used for information gathering, but they should not be used to make or rule out a referral for an assessment for ASD.	Health care professionals must have a good understanding of the different forms of expression of ASD symptomatology across developmental stages and the symptomatology of common coexisting and alternative conditions.	- Primary healthcare providers are clinicians who provide the first point of contact within the health system for community members with health concerns. While most Australians will receive primary health care through their general practitioner (GP), primary healthcare providers may also be nurses (including general practice nurses, community nurses and nurse practitioners), allied health professionals, midwives, pharmacists, dentists and Aboriginal health workers<br>- It is suggested that a referral for an assessment of ASD concerns be initiated by a primary healthcare provider. This individual's professional discipline may differ between private and public healthcare settings, but they may need to meet specific professional requirements (e.g. be a general practitioner) to meet certain

	NICE	SIGN-HIS	NZ MOH	ACRC
	interventions provided to the child or young person.<br>- During the autism diagnostic assessment, consider any potential risk of harm to, and from, the child or young person and take appropriate action.			funding conditions, such as for Medicare.<br>- It is recommended that the primary healthcare provider has received formal professional training in typical child development and the signs and/or symptoms of common neurodevelopmental and behavioral conditions, including those associated with ASD, as well as common co-occurring and differential diagnosis conditions.<br>- If the primary healthcare professional administers clinical assessments as part of the process for initiating a referral for an assessment of ASD concerns, they should have training and expertise in administering these assessments.
DIAGNOSIS				
**Assessment setting**	1.1.2 The overall configuration and development of local	Not mentioned	Not mentioned	- It is recommended that a clinic setting be considered an

	NICE	SIGN-HIS	NZ MOH	ACRC
	services (including health, mental health, learning disability, education and social care services) for autistic children and young people, should be coordinated by a local autism multiagency strategy group (for autistic people of all ages) in line with the NICE guidelines on autism in children and young people (covering identification and diagnosis) and autism in adults.<br>-The assessment, management and coordination of care for autistic children and young people should be provided through local specialist community-based multidisciplinary teams ('local autism teams') which should include professionals from health, mental health, learning disability, education and social care services in line with the			appropriate, but not essential, venue for an assessment of ASD concerns.<br>- It is recommended that information about an individual's presentation in all community settings relevant to their daily life be collected.<br>- It is recommended that information about an individual's presentation in community settings be obtained by one or more members of the Assessment Team through a combination of:<br>a. direct observation in the community setting<br>b. asking the client(s) about behavior in the community setting during an interview or through a questionnaire or survey<br>c. observation of video recordings of the individual in the community setting that have been recorded and supplied by the client or other professional(s) with the client's permission

	NICE	SIGN-HIS	NZ MOH	ACRC
	NICE guidelines on autism in children and young people (covering identification and diagnosis) and autism in adults.<br>- Local autism teams should ensure that every child or young person diagnosed with autism has a case manager or key worker to manage and coordinate treatment, care, support and transition to adult care in line with the NICE guideline on autism in children and young people (covering identification and diagnosis).<br>- Local autism teams should provide (or organize) the interventions and care recommended in this guideline for autistic children and young people who have particular needs, including: looked-after children and young people • those from immigrant groups, those with regression in skills,			d. verbal or written communication about the client's behavior in the community setting from other professional(s).<br>- It is recommended that the Single Clinician Diagnostic Evaluation take place in a setting that allows the clinician to make direct observation of symptoms. This may be in a clinic or community setting, and may be supplemented by telehealth. Information is to be collected about the client's level of functioning in all relevant community settings, though it is not essential for the clinician to make direct observations within these locations.<br>- The Comprehensive Needs Assessment comprises an Assessment of Functioning and a Medical Evaluation, with each component having different recommendations in terms of the professionals involved,

	NICE	SIGN-HIS	NZ MOH	ACRC
	those with coexisting conditions such as: severe visual and hearing impairments, other medical problems including epilepsy or sleep and elimination problems, motor disorders including cerebral palsy, intellectual disability, severe communication impairment, including lack of spoken language, or complex language disorders, and mental health problems.<br>- Local autism teams should have a key role in the delivery and coordination of: specialist care and interventions for autistic children and young people, including those living in specialist residential accommodation, advice, training and support for other health and social care professionals and staff (including in residential and			information collection techniques and observation settings.<br>- It is recommended that an Assessment of Functioning be conducted by a clinician or clinicians meeting one of the following eligibility criteria:<br>a. medical practitioner who holds general or specialist registration with the Medical Board of Australia<br>b. nurse practitioner who holds general registration with the Nursing and Midwifery Board of Australia and is endorsed as a nurse practitioner or as a registered nurse with relevant experience as a clinical nurse specialist/consultant and is practicing under appropriate medical supervision<br>c. occupational therapist who holds registration with the Occupational Therapy Board of Australia

	NICE	SIGN-HIS	NZ MOH	ACRC
	community settings) who may be involved in the care of autistic children and young people, advice and interventions to promote functional adaptive skills including communication and daily living skills, assessing and managing behavior that challenges, assessing and managing coexisting conditions, reassessing needs throughout childhood and adolescence, taking particular account of transition to adult services, supporting access to leisure and enjoyable activities, supporting access to and maintaining contact with educational, housing and employment services, providing support for families (including siblings) and careers, including offering short breaks and other respite care, producing local protocols			d. psychologist who holds general registration, with or without a practice endorsement, with the Psychology Board of Australia<br>e. social worker who is eligible to be a member of the Australian Association of Social Workers<br>f. speech pathologist who is eligible to be a Certified Practicing Member of Speech Pathology Australia.<br>- It is recommended that an Assessment of Functioning be conducted by a clinician who, in addition to the relevant training and expertise required by all members of the Assessment Team, has relevant training and expertise in:<br>a. the impact of the signs and/or symptoms of ASD and other neurodevelopmental disorders on daily functioning and participation in age-appropriate activities<br>b. the evaluation of the abilities, challenges, strengths,

	NICE	SIGN-HIS	NZ MOH	ACRC
	for: information sharing, communication and collaborative working among healthcare, education and social care services, including arrangements for transition to adult services, shared care arrangements with primary care providers and ensuring that clear lines of communication between primary and secondary care are maintained.<br>- Refer autistic children and young people to a regional or national autism service if there is a lack of: local skills and competencies needed to provide interventions and care for a child or young person with a complex coexisting condition, such as a severe sensory or motor impairment or mental health problem, or response to the therapeutic			environmental context and support needs of individuals with ASD and other neurodevelopmental disorders (along with those of their caregivers and support people).<br>- It is recommended that information be collected during an Assessment of Functioning on the following topics:<br>a. medical and health history, including any existing diagnoses<br>b. family history and family functioning<br>c. language/s used at home and level of written/spoken proficiency in English and any other home language<br>d. developmental and educational history<br>e. ASD-specific signs and/or symptoms<br>f. other relevant signs and/or symptoms<br>g. developmental and functioning abilities/impairments across a

	NICE	SIGN-HIS	NZ MOH	ACRC
	interventions provided by the local autism team.<br>- Health and social care professionals working with autistic children and young people in any setting should receive training in autism awareness and skills in managing autism, which should include: the nature and course of autism, the nature and course of behavior that challenges in autistic children and young people, recognition of common coexisting conditions, including: mental health problems such as anxiety and depression, physical health problems such as epilepsy, sleep problems , other neurodevelopmental conditions such as attention deficit hyperactivity disorder (ADHD), the importance of key transition points, such as changing schools or health or			broad range of domains (e.g. cognitive, language, social-emotional, motor and adaptive behavior)<br>h. activity-related and character strengths<br>i. environmental facilitators and barriers<br>j. observed and expressed support needs.<br>- It is recommended that information be collected during an Assessment of Functioning through a variety of means, including:<br>a. file review of existing assessment reports<br>b. interview with the client<br>c. observation of the individual undergoing assessment<br>d. administration of standardized and non-standardized assessments as required<br>e. communication with other professional(s) as required.

	NICE	SIGN-HIS	NZ MOH	ACRC
	social care services , the child or young person's experience of autism and its impact on them, the impact of autism on the family (including siblings) or careers, the impact of the social and physical environment on the child or young person, how to assess risk (including self-harm, harm to others, self-neglect, breakdown of family or residential support, exploitation or abuse by others) and develop a risk management plan, the changing needs that arise with puberty (including the child or young person's understanding of intimate relationships and related problems that may occur, for example, misunderstanding the behavior of others), how to provide individualized care and support and ensure a			- It is recommended that the use of standardized assessments that cover a broad range of developmental domains (e.g. cognitive, language, social-emotional, motor and adaptive behavior) be strongly considered for the Comprehensive Needs Assessment.<br>- It is recommended that the Assessment of Functioning take place in a setting where the client feels comfortable and confident to discuss their level of functioning and support needs.<br>- Information is to be collected about the individual's level of functioning in all relevant community settings, though it is not essential for the clinician to make direct observations at these locations.<br>- It is recommended that the Assessment of Functioning involve the following steps:

	NICE	SIGN-HIS	NZ MOH	ACRC
	consistent approach is used across all settings<br>-Take into account the physical environment in which autistic children and young people are supported and cared for.<br>- Minimize any negative impact by: providing visual supports, for example, words, pictures or symbols that are meaningful for the child or young person, making reasonable adjustments or adaptations to the amount of personal space given, considering individual sensory sensitivities to lighting, noise levels and the color of walls and furnishings.<br>- Make adjustments or adaptations to the processes of health or social care, for example, arranging appointments at the beginning or end of the day to minimize waiting time, or providing			a. the identification and prioritization of observed and expressed support needs<br>b. connection to appropriate services based on support needs where impaired functioning is identified, without the requirement for a clinical diagnosis of ASD.<br>- It is suggested that when providing information to clients regarding services that may meet their support needs, clinician(s):<br>a. disclose to the client any financial or other conflicts of interest in service recommendations<br>b. provide information regarding a range of services available, where possible.<br>- It is recommended that the Assessment of Functioning be repeated throughout the individual's life to ensure that changes to level of functioning and support needs are identified

	NICE	SIGN-HIS	NZ MOH	ACRC
	single rooms for children and young people who may need a general anesthetic in hospital (for example, for dental treatment).			and acted on in a timely manner. Further assessment can be conducted as required by clinicians engaging with the client at the particular time.<br>- It is recommended that a Medical Evaluation and investigations relevant to neurodevelopmental and behavioral disorders be conducted by a medical practitioner who holds general or specialist registration with the Medical Board of Australia.<br>- It is suggested that a medical practitioner may receive assistance in collecting information for the Medical Evaluation from a nurse practitioner who holds general registration with the Nursing and Midwifery Board of Australia and is endorsed as a nurse practitioner or as a registered nurse with relevant experience as a clinical nurse

	NICE	SIGN-HIS	NZ MOH	ACRC
				specialist/consultant, practicing under appropriate medical supervision.<br>- It is recommended that a Medical Evaluation be conducted by a clinician who, in addition to the relevant training and expertise required by all members of the Assessment Team, has relevant training and expertise in medical evaluation relevant to neurodevelopmental disorders.<br>- It is recommended that information be collected and synthesized during a Medical Evaluation on the following:<br>a. overview of topics covered in the Assessment of Functioning<br>b. neurodevelopmental and behavioral symptoms<br>c. relevant biological investigations for etiology and comorbid conditions (further testing may be indicated after Diagnostic Evaluation, e.g. chromosomal microarray)

	NICE	SIGN-HIS	NZ MOH	ACRC
				d. developmental and growth status<br>e. congenital abnormalities and dysmorphic features<br>f. neurological, general systems, skin, injury, vision and hearing status.<br>- It is recommended that information be collected during a Medical Evaluation through a variety of means, including:<br>a. file review of any relevant assessment reports<br>b. interview with the client<br>c. observation of the individual undergoing assessment<br>d. physical examination<br>e. standardized assessments<br>f. communication with other professional(s) as required.<br>- It is recommended that the Medical Evaluation take place in a private location within a clinic or community setting.<br>- It is suggested that if the Assessment of Functioning and

	NICE	SIGN-HIS	NZ MOH	ACRC
				Medical Evaluation indicate ASD is a queried diagnosis, the clinician in consultation with the client will make a referral for a Single Clinician Diagnostic Evaluation. If the client declines this referral, it is recommended this be documented by the clinician.
**Diagnostic criteria**	Include in every autism diagnostic assessment:<br>a. detailed questions about parent's or career's concerns and, if appropriate, the child's or young person's concerns details of the child's or young person's experiences of home life, education and social care a developmental history, focusing on developmental and behavioral features consistent with ICD-10 or DSM-5 criteria<br>b. assessment of social and communication skills and	- All professionals involved in diagnosing ASD in children, young people or adults should consider using the current version of either ICD or DSM. The classification system used for diagnosis should be recorded in the patient's notes.	- Experienced clinicians are usually necessary for accurate and appropriate diagnosis of autism.<br>- Clinical judgment may be aided by diagnostic guides such as DSM-IV or ICD-10 as well as assessment tools, checklists and rating scales	- It is suggested that a Single Clinician Diagnostic Evaluation be conducted by one clinician meeting at least one of the following eligibility criteria:<br>a. medical practitioner who holds specialist registration with the Medical Board of Australia in the field of community child health, general paediatrics, psychiatry or neurology<br>b. medical practitioner who holds general or specialist registration with the Medical Board of Australia and has at least six years of relevant experience, training or

	NICE	SIGN-HIS	NZ MOH	ACRC
	behaviors, focusing on features consistent with ICD-10 or DSM-5 criteria			supervision in the assessment of neurodevelopmental and behavioural disorders<br>c. psychologist who holds general registration with the Psychology Board of Australia and practice endorsement in clinical psychology, educational/developmental psychology or neuropsychology.<br>- It is recommended that a Single Clinician Diagnostic Evaluation be conducted by a clinician who, in addition to the expertise required by all members of the Assessment Team, has relevant training and expertise in the following areas:<br>a. clinical reasoning in weighing evidence, and performing diagnostic formulations and decisions<br>b. signs and symptoms associated with common co-occurring or differential diagnosis conditions<br>c. the criteria for ASD and co-occurring or differential diagnosis

	NICE	SIGN-HIS	NZ MOH	ACRC
				conditions described by the current version of international diagnostic manuals (e.g. DSM and/or ICD).<br>- It is recommended that the Single Clinician obtain and maintain the additional skills and expertise listed in Recommendation 38 through peer observation, peer supervision and peer mentoring. Formal training courses and/or further qualifications may supplement these peer learning approaches.<br>- It is suggested that a Single Clinician Diagnostic Evaluation involve the collection of information from at least one other clinician from a different discipline or specialty to the Single Clinician, if information from at least one clinician from a different discipline has not yet been obtained.

	NICE	SIGN-HIS	NZ MOH	ACRC
				- It is recommended that information be collected during a Single Clinician Diagnostic Evaluation on the following:<br>a. overview of topics covered in the Comprehensive Needs Assessment<br>b. signs and/or symptoms specified in diagnostic criteria for ASD and potential co-occurring and/or differential conditions<br>c. biological, personal and environmental factors relevant to the individual.<br>- It is recommended that information be collected during a Single Clinician Diagnostic Evaluation through a variety of means, including:<br>a. review of documentation from the Comprehensive Needs Assessment<br>b. communication with clinicians who conducted the Comprehensive Needs Assessment

	NICE	SIGN-HIS	NZ MOH	ACRC
				c.file review of any additional assessment reports<br>d. interview with the client;<br>e.observation of the individual undergoing assessment<br>f. communication with other professional(s) as required.<br>- It is suggested that ASD-specific assessments not be used as a substitute for clinical judgement in diagnostic decision-making, nor as the sole investigation on which an ASD diagnosis is based, though they may provide considerable assistance in the direct observation of ASD symptoms. Their use in an assessment of ASD concerns is at the discretion of the Single Clinician.<br>- It is recommended that the clinician conducting the Single Clinician Diagnostic Evaluation use their clinical judgement to reach their diagnostic decision by:<br>a. taking into account all information collected in the

	NICE	SIGN-HIS	NZ MOH	ACRC
				Comprehensive Needs Assessment and Single Clinician Diagnostic Evaluation, in the context of a biopsychosocial framework<br>b. integrating and weighing the available evidence against each diagnostic criterion (according to the current version of the DSM or ICD)<br>c. testing alternative explanations for symptoms that may warrant co-occurring or differential diagnosis or alternative clinical pathways<br>d. considering whether sufficient information is available to make a diagnostic decision with high confidence without progressing to a Consensus Team Diagnostic Evaluation.<br><br>- It is recommended that any new support needs identified at the Single Clinician Diagnostic Evaluation be documented,

	NICE	SIGN-HIS	NZ MOH	ACRC
				communicated to the client and, if appropriate, communicated to the client's current support services (with the client's permission). If the client is not receiving any support services, it is recommended that they be connected to appropriate services based on support needs, without the requirement for a clinical diagnosis of ASD.<br>- It is suggested that the clinician who conducted the Single Clinician Diagnostic Evaluation invite additional clinician(s) as required to participate in the Consensus Team Diagnostic Evaluation, based on the match between professional expertise and the area(s) of diagnostic uncertainty identified during the Single Clinician Diagnostic Evaluation.<br>- It is recommended that a Consensus Team Diagnostic Evaluation include at least one

	NICE	SIGN-HIS	NZ MOH	ACRC
				additional clinician who meets at least one of the following eligibility criteria:<br>a. medical practitioner who holds specialist registration with the Medical Board of Australia in the field of community child health, general paediatrics, psychiatry or neurology<br>b. medical practitioner who holds general or specialist registration with the Medical Board of Australia and has at least six years of relevant experience, training or supervision in the assessment of neurodevelopmental disorders<br>c. occupational therapist who holds registration with the Occupational Therapy Board of Australia<br>d. psychologist who holds general registration, with or without a practice endorsement, with the Psychology Board of Australia<br>e. speech pathologist who is eligible to be a Certified Practicing

	NICE	SIGN-HIS	NZ MOH	ACRC
				Member of Speech Pathology Australia.<br>- It is recommended that a Consensus Team Diagnostic Evaluation be conducted by clinicians who, in addition to the relevant training and expertise required by all members of the Assessment Team, have relevant training and expertise in all the following areas:<br>a. clinical reasoning in weighing evidence, performing diagnostic formulations and making diagnostic decisions<br>b. signs and symptoms associated with common co-occurring or differential diagnosis conditions<br>c. the criteria for ASD and co-occurring or differential diagnosis conditions described by the current version of international diagnostic manuals (e.g. DSM and/or ICD).<br>- It is recommended that members of the Consensus

	NICE	SIGN-HIS	NZ MOH	ACRC
				Diagnosis Team obtain and maintain the additional skills and expertise listed in Recommendation 49 through peer observation, peer supervision and peer mentoring. Formal training courses and/or further qualifications may supplement these peer learning approaches.<br>- It is recommended that information be collected during a Consensus Team Diagnostic Evaluation on the following:<br>a. overview of topics covered in the Comprehensive Needs Assessment and Single Clinician Diagnostic Evaluation<br>b. additional information to further appraise behavioral symptoms specified in diagnostic criteria for ASD and potential co-occurring and/or differential diagnosis conditions<br>c. further exploration of biological, personal and

	NICE	SIGN-HIS	NZ MOH	ACRC
				environmental factors relevant to the individual.<br>- It is recommended that information be collected during a Consensus Team Diagnostic Evaluation through a variety of means, including:<br>a. review of documentation from the Comprehensive Needs Assessment and Single Clinician Diagnostic Evaluation<br>b. communication with clinicians who conducted the Comprehensive Needs Assessment and Single Clinician Diagnostic Evaluation<br>c. file review of any additional assessment reports<br>d. interview with the client as required<br>e. observation of the individual undergoing assessment<br>f. administration of standardized and non-standardized assessments as required

	NICE	SIGN-HIS	NZ MOH	ACRC
				g. communication with other professional(s) as required.<br>- It is suggested that ASD-specific assessments not be used as a substitute for clinical judgement in diagnostic decision-making, though they may provide considerable assistance in the direct observation of ASD symptoms, and their use in an assessment of ASD concerns be at the discretion of the Consensus Diagnosis Team.<br>- It is recommended that the Consensus Team Diagnostic Evaluation take place in a setting that allows the clinician to assess how symptoms manifest in a variety of contexts relevant to the client:<br>a. this may be in a combination of clinic and community settings, which may be supplemented by information collected in a telehealth setting.

	NICE	SIGN-HIS	NZ MOH	ACRC
				b. this information may be obtained through communication with the client and/or other professionals, but direct observations by member(s) of the Consensus Diagnosis Team within some of these community settings is suggested where possible.<br>- It is recommended that clinicians conducting the Consensus Team Diagnostic Evaluation use their clinical judgement to reach a consensus diagnostic decision by:<br>a. taking into account all information collected during all stages of assessments, in the context of a biopsychosocial framework<br>b. integrating and weighing the available evidence against each diagnostic criterion (according to the current version of the DSM or ICD)<br>c. testing alternative explanations for signs and/or symptoms that may warrant co-occurring or

	NICE	SIGN-HIS	NZ MOH	ACRC
				differential diagnosis or alternative clinical pathways<br>d. considering if sufficient information is available to make a diagnostic decision with high confidence<br>e. discussing the evidence until each member of the Consensus Diagnosis Team agrees on the same diagnostic outcome.<br>- It is recommended that any new support needs identified at the Consensus Team Diagnostic Evaluation be documented, communicated to the client and, if appropriate, communicated to the client's current support services (with the client's permission). If the client is not receiving any support services, it is recommended that they be connected to appropriate services based on support needs, without the requirement for a clinical diagnosis of ASD.

	NICE	SIGN-HIS	NZ MOH	ACRC
**Timing of diagnosis**	- If young people present at the time of transition to adult services, the autism team should consider carrying out the autism diagnostic assessment jointly with the adult autism team, regardless of the young person's intellectual ability.	- ASD should be part of the differential diagnosis for preschool children displaying absence of age appropriate developmental features, as typical ASD behaviors may not be obvious in this age group. GPP<br>- Regardless of the findings of any earlier assessments, referral for further assessment for ASD should be considered at any age.	- Age of detection/diagnosis of all developmental problems, including ASD as a specified disorder, should be audited.	Not mentioned
**Initial Assessment**	- Consider the possibility of autism if there are concerns about development or behavior, but be aware that there may be other explanations for individual signs and symptoms.<br>- Always take parents' or careers' concerns and, if appropriate, the child's or	- If, on the basis of initial assessment, it is suspected that the individual may have ASD, they should be referred for specialist assessment.	- The initial assessment of children may be undertaken by an individual practitioner. If there are ongoing concerns, a multidisciplinary assessment is recommended.	- It is suggested that the primary healthcare provider obtain information about ASD signs and/or symptoms in a structured way through client report and/or observation, along with administering a standardized developmental screening measure when age appropriate.

	NICE	SIGN-HIS	NZ MOH	ACRC
	young person's concerns, about behavior or development seriously, even if these are not shared by others.<br>- When considering the possibility of autism and whether to refer a child or young person to the autism team, be critical about your professional competence and seek advice from a colleague if in doubt about the next step.<br>- Do not rule out autism if the exact features described in the tables are not evident; they should be used for guidance, but do not include all possible manifestations of autism.<br>- When considering the possibility of autism, be aware that:<br>a. signs and symptoms should be seen in the context of the child's or young person's overall development		- Preferably, a multidisciplinary team of health care practitioners experienced in ASD should undertake diagnostic assessment of young people and adults suspected of having ASD. In the absence of an assessment team, a health care practitioner trained and highly experienced in ASD may undertake diagnostic assessment.<br>- Formal pathways for diagnostic assessment of young people and adults should be developed.<br><br>- Diagnostic assessment of young people and adults should be comprehensive	- It is recommended that the primary healthcare provider initiate an assessment of ASD concerns by discussing and obtaining the client's consent for the referral and then providing a written referral to the Assessment Team, including the reasons for referral and necessary information to efficiently commence the process.<br>- It is suggested that, on receiving the referral, a nominated clinician (or their delegate) from the Assessment Team explain the process for assessing ASD concerns to the client, book the initial appointment (ideally within three months of referral), collate existing documents that may assist with the assessment (e.g. previously administered client questionnaires, reports from treating clinicians and school records) and give the client details

	NICE	SIGN-HIS	NZ MOH	ACRC
	b. signs and symptoms will not always have been recognized by parents, careers, children or young people themselves or by other professional<br>c. when older children or young people present for the first time with possible autism, signs or symptoms may have previously been masked by the child or young person's coping mechanisms and/or a supportive environment<br>d. it is necessary to take account of cultural variation, but do not assume that language delay is accounted for because English is not the family's first language or by early hearing difficulties<br>c. autism may be missed in children or young people with a learning (intellectual) disability		(covering all areas listed below), and involve the person concerned in interview and observation<br><br>- Standardized ASD assessment interviews and schedules should be used - Test users should ensure that they are aware of the validity, reliability and appropriateness of tests when assessing people with ASD and take these limitations into account when forming opinions and reporting results.<br><br>- The intellectual, adaptive and cognitive skills associated with ASD should be seriously considered and, where	for how to contact the Assessment Team.

	NICE	SIGN-HIS	NZ MOH	ACRC
	d. autism may be missed in children or young people who are verbally able<br>e. autism may be under-diagnosed in girls<br>f. important information about early development may not be readily available for some children and young people, for example looked-after children and those in the criminal justice system<br>g. signs and symptoms may not be accounted for by disruptive home experiences or parental or career mental or physical illness.<br>-When considering the possibility of autism, ask about the child or young person's use and understanding of their first language.<br>- Do not rule out autism because of:		possible and appropriate, formally assessed.	

	NICE	SIGN-HIS	NZ MOH	ACRC
	a. good eye contact, smiling and showing affection to family members<br>b. To help identify the signs and symptoms of possible autism.<br>c. do not rule out autism if the exact features described in the tables are not evident; they should be used for guidance, but do not include all possible manifestations of autism.<br>- Discuss developmental or behavioral concerns about a child or young adults:<br>a. factors associated with an increased prevalence of autism<br>b. the likelihood of an alternative diagnosis.<br>- If you have concerns about development or behavior but are not sure whether the signs and/or symptoms suggest autism, consider:			

	NICE	SIGN-HIS	NZ MOH	ACRC
	a. consulting a member of the autism team who can provide advice to help you decide if a referral to the autism team is necessary<br>b. referring to another service. That service can then refer to the autism team if necessary.<br>- Be aware that tools to identify children and young people with an increased likelihood of autism may be useful in gathering information about signs and symptoms of autism in a structured way but are not essential and should not be used to make or rule out a diagnosis of autism. Also be aware that:<br>a. positive score on tools to identify an increased likelihood of autism may support a decision to refer but can also be for reasons other than autism			

	NICE	SIGN-HIS	NZ MOH	ACRC
	b. negative score does not rule out autism.			
**Special Assessment**	- If there are discrepancies during the autism diagnostic assessment between reported signs or symptoms and the findings of the autism observation in the clinical setting, consider:<br>a. gathering additional information from other sources and/or<br>b. carrying out further autism-specific observations in different settings, such as the school, nursery, other social setting or at home.<br>- Use information from all sources, together with clinical judgment, to diagnose autism based on ICD-10 or DSM-5 criteria.<br>- Do not rely on any autism-specific diagnostic tool alone to diagnose autism.	- A diagnostic assessment, alongside a profile of the individual's strengths and weaknesses, carried out by a multidisciplinary team which has the skills and experience to undertake the assessments, should be considered as the optimum approach for individuals suspected of having ASD.<br>GPP<br>-The use of different professional groups in the assessment process is recommended as it may identify different aspects of ASD and aid accurate diagnosis.<br>- Specialist assessment should involve a history-taking element, a clinical observation/assessment element, and the obtaining of	All children suspected of having ASD or another developmental problem should have an audiology assessment.	- It is recommended that in circumstances where a clinician with the professional background and assessment expertise prerequisites to being a member of a Consensus Diagnosis Team is not present in the local community, a partnership between local clinicians and an assessment team in another location be facilitated through telehealth or other methods.<br>- It is recommended that before conducting an assessment of ASD concerns, professionals within the Assessment Team have a good understanding of the support services available for individuals in the local regional or remote community.<br>- It is recommended that all members of the Assessment Team have a good understanding of complex psychosocial factors

	NICE	SIGN-HIS	NZ MOH	ACRC
	- Be aware that in some children and young people there may be uncertainty about the diagnosis of autism, particularly in:<br>a. children younger than 24 months<br>b. Children or young people with a developmental age of less than 18 months<br>children or young people for whom there is a lack of available information about their early life (for example, some looked-after or adopted children) older teenagers' children or young people with a complex coexisting mental health disorder (for example ADHD, conduct disorder, a possible attachment disorder), sensory impairment (for example severe hearing or visual impairment), or a motor disorder such as cerebral palsy.	wider contextual and functional information.<br>- Specialist assessment should be available for any individuals who need it. Specialist teams should assess if their service is being used equitably.<br>- Apparent inequalities should be investigated and addressed. An assessment of mental health needs, well-being and risk should be considered for all individuals with ASD presenting to any agency.		and their potential impact on the individual's behavioral presentation and needs.

	NICE	SIGN-HIS	NZ MOH	ACRC
	- Be aware that some children and young people will have features of behavior that are seen in the autism spectrum but do not reach the ICD-10 or DSM-5 diagnostic criteria for definitive diagnosis. Based on their profile, consider referring to appropriate services.<br>- If the outcome of the autism diagnostic assessment clearly indicates that the child or young person does not have autism, consider referring them to appropriate services based on their profile.<br>- Consider whether the child or young person may have any of the following as a coexisting condition, and if suspected carry out appropriate assessments and referrals:<br>a. Mental and behavior problems and disorders: ADHD, anxiety disorders and phobias, mood disorders,			

	NICE	SIGN-HIS	NZ MOH	ACRC
	oppositional defiant behavior, tics or Tourette syndrome, OCD, self-injurious behavior.<br>b. Neurodevelopmental problems and disorders: global delay or a learning (intellectual) disability, motor coordination problems or DCD, academic learning problems, for example in literacy or numeracy, speech and language disorder.<br>- Perform a general physical examination and look specifically for:<br>a. skin stigmata of neurofibromatosis or tuberous sclerosis using a Wood's light, signs of injury, for example self-harm child maltreatment<br>b. congenital anomalies and dysmorphic features including macrocephaly or microcephaly.<br>- Consider the following differential diagnoses for			

	NICE	SIGN-HIS	NZ MOH	ACRC
	autism and whether specific assessments are needed to help interpret the autism history and observations:<br><br>a. Neurodevelopmental disorders: specific language delay or disorder<br>b. a learning (intellectual) disability or global developmental delay - developmental coordination disorder (DCD).<br>c. Medical or genetic problems and disorders: epilepsy and epileptic encephalopathy, chromosome disorders, genetic abnormalities, including fragile X, tuberous sclerosis, muscular dystrophy, neurofibromatosis.<br>d. Functional problems and disorders: feeding problems, including restricted diets, urinary incontinence or enuresis, constipation, altered			

	NICE	SIGN-HIS	NZ MOH	ACRC
	bowel habit, fecal incontinence or encopresis, sleep disturbances, vision or hearing impairment.<br>- Be aware that in children and young people with communication difficulties it may be difficult to recognize functional problems or mental health problems.<br>- If there is uncertainty after the autism diagnostic assessment about the diagnosis, consider keeping the child or young person under review, taking into account any new information.			
**Component of Assessment Assessment of language, cognitive, adaptive, motor, and sensory**	- Include in every autism diagnostic assessment:<br>a. detailed questions about parent's or career's concerns and, if appropriate, the child's or young person's concerns details of the child's or young person's experiences of home	- All children and young people with ASD should have a comprehensive evaluation of their speech and language and communication skills, which should inform intervention.<br>- Healthcare professionals should note that an	- Standardized ASD assessment interviews and schedules should be used. The intellectual, adaptive and cognitive skills associated with ASD should be seriously considered and, where	- It is recommended that all members of the Assessment Team consider the individual's behavioral presentation and needs in comparison to other individuals of the same chronological and developmental age.

	NICE	SIGN-HIS	NZ MOH	ACRC
	life, education and social care a developmental history, focusing on developmental and behavioral features consistent with ICD-10 or DSM-5 criteria<br>b. assessment of social and communication skills and behaviors, focusing on features consistent with ICD-10 or DSM-5 criteria<br>c. a medical history, including prenatal, perinatal and family history, and past and current health conditions a physical examination<br>d. consideration of the differential diagnosis development of a profile of the child's or young person's strengths, skills, impairments and needs that can be used to create a needs-based management plan, taking into account family and educational context.	individual's level of comprehension may be at a lower developmental level than that suggested by their expressive language skills.<br>- Individuals with ASD should be considered for assessment of intellectual, neuropsychological and adaptive functioning.<br>MOTOR AND SENSORY SKILLS<br><br>- Sensory behaviors should be taken into account when profiling the needs of individuals with ASD.<br>- Occupational therapy and physiotherapy assessments should be considered where relevant.	possible and appropriate, formally assessed.	- It is suggested that all members of the Assessment Team consider the individual's cognitive/intellectual abilities and verbal language level when choosing standardized assessments and determining the individual's ability to provide valid consent.<br>- It is recommended that all members of the Assessment Team consider the individual's behavioral presentation and needs in comparison to other individuals of the same gender, and be aware of how ASD may manifest differently in males and females.<br>- There is accumulating evidence that being transgender or gender diverse is more common in children, adolescents and adults on the autism spectrum compared to the broader population.<br>- It is suggested that all members of the Assessment Team have a

	NICE	SIGN-HIS	NZ MOH	ACRC
	e. communication of assessment findings to the parent or career and, if appropriate, the child or young person.<br>- Perform a general physical examination and look specifically for:<br>a. skin stigmata of neurofibromatosis or tuberous sclerosis using a Wood's light<br>b. signs of injury, for example self-harm or child maltreatment<br>c. congenital anomalies and dysmorphic features including macrocephaly or microcephaly.			good understanding of gender diversity and its potential impact on the individual's behavioural presentation and needs.<br>- It is recommended that all members of the Assessment Team consider the racial or ethnic background of the individual, including Aboriginal people, and how cultural factors relevant to the individual and their caregiver(s) may guide or influence the process of assessing ASD concerns.<br>- For Aboriginal people in particular, it is recommended that the role of the family, extended family and community be acknowledged and empowered by identifying attitudes and beliefs that the individual and family have surrounding ASD.<br>- It is recommended that a client receive cultural support from a community member or appropriate professional (e.g. Aboriginal health worker) if this is

	NICE	SIGN-HIS	NZ MOH	ACRC
				requested or identified as potentially beneficial during the assessment of ASD concerns.<br>- It is suggested that this support be available from the receipt of referral through to the communication of assessment findings and connection to support services.<br>- It is recommended that interpreter services and translated educational materials be made available for all clients from a non-English speaking background (including those who speak an Aboriginal language).
**Differential diagnosis**	- Consider the following differential diagnoses for autism and whether specific assessments are needed to help interpret the autism history and observations:<br>a. Neurodevelopmental disorders:<br>i) specific language delay or disorder	- Healthcare professionals should be aware of the need to routinely check for coexisting problems in children and young people with ASD. Where necessary, detailed assessment should be carried out to accurately identify and manage coexisting problems.	- Differential diagnosis must be covered during diagnostic assessment.<br>- Differential diagnosis must be thorough and cover all conditions commonly confused with ASD and those known to coexist with ASD.	- The behavioral signs and/or symptoms that define ASD are often observed in individuals with other clinical conditions, and individuals on the autism spectrum often present with signs and/or symptoms that are characteristic of other clinical conditions.

	NICE	SIGN-HIS	NZ MOH	ACRC
	ii) a learning (intellectual) disability or global developmental delay<br>iii) developmental coordination disorder (DCD).<br>b. Mental and behavioral disorders:<br>i) attention deficit hyperactivity disorder (ADHD)<br>ii) mood disorder<br>iii) anxiety disorder<br>iv)attachment disorders<br>v) oppositional defiant disorder (ODD)<br>vi) conduct disorder<br>vii) obsessive compulsive disorder (OCD)<br>viii) psychosis.<br>C. Conditions in which there is developmental regression: i) Rett syndrome<br>ii) epileptic encephalopathy.<br>iii) Other conditions: severe hearing impairment, severe visual impairment,	- Healthcare professionals should recognize that children and young people with ASD may also have additional developmental disorders, medical problems or emotional difficulties/disorders and should have access to the same range of therapeutic interventions as any other child.	-Health care professionals must have a good understanding of the different forms of expression of ASD symptomatology across developmental stages and the symptomatology of common coexisting and alternative conditions	- It is recommended that, at each stage of the Diagnostic Evaluation, the clinicians collect and evaluate information to consider the full range of clinical explanations for the presentation of signs and/or symptoms, and test these possible explanations against the evidence for an ASD diagnosis in the context of other differential and co-occurring diagnoses.<br>- It is recommended that members of the Assessment Team be highly familiar with the range of differential diagnoses for ASD. Clinicians without the clinical qualifications or expertise to adequately evaluate potential differential diagnoses for a given individual should not undertake the assessment of ASD concerns.

	NICE	SIGN-HIS	NZ MOH	ACRC
	maltreatment, and selective mutism.<br>- Consider which assessments are needed to construct a profile for each child or young person:<br>a. intellectual ability and learning style<br>academic skills<br>b. speech, language and communication<br>c. fine and gross motor skills<br>d. adaptive behavior (including self-help skills)<br>e. mental and emotional health (including self-esteem)<br>f. physical health and nutrition<br>h. sensory sensitivities<br>g. behavior likely to affect day-to-day functioning and social participation<br>j. socialization skills.			
**Medical investigations**	- Do not routinely perform any medical investigations as part of an autism diagnostic	- Where clinically relevant, the need for the following should	- Pre-treatment assessments should gather detailed	Not mentioned

	NICE	SIGN-HIS	NZ MOH	ACRC
	assessment, but consider the following in individual circumstances and based on physical examination, clinical judgment and the child or young person's profile: genetic tests, as recommended by your regional genetics center, if there are specific dysmorphic features, congenital anomalies and/or evidence of a learning (intellectual) disability, electroencephalography if there is suspicion of epilepsy.	be reviewed for all individuals with ASD:<br>a. examination of physical status, with particular attention to neurological and dysmorphic features chromosomal microarray<br>b. examination of audiological status<br>c. investigations to rule out recognized etiologies of ASD (e.g. tuberous sclerosis).<br>- Advice on further testing should be sought from the local genetics service.	information on behavioral, emotional and mental health difficulties, address differential diagnosis, screen for medical conditions and address environmental issues.	
**Disclosure of the results of diagnostic assessment**	After the autism diagnostic assessment discuss the findings, including the profile, sensitively, in person and without delay with the parents or careers and, if appropriate, the child or young person. Explain the basis of conclusions even if the diagnosis of autism was not reached.	- Professionals should offer individuals, parents and careers good-quality written information and an opportunity to ask questions when sharing information about the individual with ASD.<br>- Information should be provided in an accessible and understandable form.	- Formulation is the necessary next step from assessment.<br>- Clarity of diagnosis should be the goal of assessment and formulation.<br>- In situations when diagnostic clarity is not possible, an action plan should	- It is recommended that the findings of the assessment of ASD concerns be communicated to the client by the Single Clinician and/or at least one member of the Consensus Diagnosis Team in a comprehensive and understandable way through a face-to-face meeting (or via a telehealth setting) and a written report. This will

	NICE	SIGN-HIS	NZ MOH	ACRC
	- Use recognized good practice when sharing a diagnosis with parents, careers, children and young people.<br>- For children and young people with a diagnosis of autism, discuss and share information with parents or careers and, if appropriate, the child or young person, to explain: what autism is how autism is likely to affect the child or young person's development and function.<br>- Provide parents or careers and, if appropriate, the child or young person, with a written report of the autism diagnostic assessment. This should explain the findings of the assessment and the reasons for the conclusions drawn.<br>- Share information, including the written report of the		be developed to attend to areas of complexity and confusion.<br>- All diagnostic assessments should include a detailed written report covering the person's strengths and weaknesses, developmental course, ASD symptoms, recommendations for intervention and information on support networks.<br>- Disclosure of diagnosis of older teens and adults and decisions about the involvement of family and whānau or support people should take into consideration the wishes of the person concerned, privacy issues and their support needs.	ideally occur within three months of the first assessment appointment, or earlier in line with the clinician's existing professional standards.<br>- Findings of the assessment of ASD concerns should be shared only with relevant stakeholders, such as the referrer, caregivers / support people, service providers or funding agencies, with the expressed consent of the client.

	NICE	SIGN-HIS	NZ MOH	ACRC
	diagnostic assessment, with the GP.<br>- With parental or career consent and, if appropriate, the consent of the child or young person, share information with key professionals involved in the child's or young person's care, including those in education and social care.<br>- With parental or career consent and, if appropriate, the consent of the child or young person, make the profile available to professionals in education (for example, through a school visit by a member of the autism team) and, if appropriate, social care. This is so it can contribute to the child or young person's individual education plan and needs-based management plan.		- Information on ASD and support services should be available at all diagnostic disclosure interviews and through health and disability services.<br>- Sources of post-diagnosis support should be identified for the person with ASD	

	NICE	SIGN-HIS	NZ MOH	ACRC
	- For children and young people with a diagnosis of autism, offer a follow-up appointment with an appropriate member of the autism team within 6 weeks of the end of the autism assessment for further discussion (for example about the conclusions of the assessment and the implications for the child or young person).<br>- For children and young people with a diagnosis of autism, discuss with parents or careers the risk of autism occurring in siblings and future children.			
**Information and support for families**	- Provide individual information on support available locally for parents, careers, and autistic children and young people, according to the family's needs. This may include:	- The information shared should relate to the individual's particular ASD presentation.<br>- People with ASD and their families/careers require support and high-quality	- The values, knowledge, preferences and cultural perspectives of the family/<br>whānau should be respected and evident in services and resources.	It is recommended that findings of the assessment of ASD concerns be communicated to the client by the Single Clinician and/or at least one member of the Consensus Diagnosis Team in a comprehensive and

	NICE	SIGN-HIS	NZ MOH	ACRC
	contact details for:<br>a. local and national support organizations (who may provide, for example, an opportunity to meet other families with experience of autism, or information about specific courses for parents and carers and/or young people)<br>b. organizations that can provide advice on welfare benefits<br>c. organizations that can provide information on educational support and social care<br>information to help prepare for the future, for example transition to adult services.<br>- Make arrangements to support autistic children and young people and their family and careers during times of increased need, including major life changes such as	verbal and written information at time of diagnosis. This should include a written report of the outcome of the various assessments and the final diagnosis. Copies of the letters sent to the various professionals who have been asked to assess their child or the adult may also be included.<br>- Professionals involved in sharing of an ASD diagnosis and information provision should receive ongoing education and training.<br>- Individuals with ASD and their parents, relatives or carers should be encouraged to continue to learn about ASD and about any useful interventions and support.	- The stress experienced by the families and whānau of children with ASD should be acknowledged.<br>- The value of parent-led support networks should be recognised in helping parents to deal with the issues that they are facing following diagnosis and<br>in supporting access to information.<br>-Teachers and other professionals should collect and appreciate the unique information about the child, which is held by the parent. This information should be incorporated into the planning of the child's education programme.	understandable way through a face-to-face meeting (or via a telehealth setting) and a written report. This will ideally occur within three months of the first assessment appointment, or earlier in line with the clinician's existing professional standards. Findings of the assessment of ASD concerns should only be shared only with relevant stakeholders, such as the referrer, caregivers / support people, service providers or funding agencies, with the expressed consent of the client. [Consensus-based Recommendation, Grade 1]<br><br>Recommendation 58<br>It is recommended that the findings of the assessment conveyed to a client at a meeting (or meetings) and in a written report (or reports) include the following information:

	NICE	SIGN-HIS	NZ MOH	ACRC
	puberty, starting or changing schools, or the birth of a sibling.<br>- Explore with autistic children and young people, and their families and careers, whether they want to be involved in shared decision-making and continue to explore these issues at regular intervals. If children and young people express interest, offer a collaborative approach to treatment and care that takes their preferences into account.<br>- Ensure that all autistic children and young people have full access to health and social care services, including mental health services, regardless of their intellectual ability or any coexisting diagnosis.<br>- Offer all families (including siblings) and careers verbal		- The parents' role in interventions should be respectfully negotiated.<br>- Planning and evaluation of interventions should always take into account both family/whānau and child variables and outcomes.<br>- ASD-related counselling and/or advocacy services and education should be available to all family members and carers.<br>- Further research is needed to identify the needs of children parented by people with ASD and the needs of parents with ASD.	– clear confirmation of the diagnostic outcome and a rationale for the diagnostic decision<br>– the diagnostic criteria utilized (e.g. DSM-5 or ICD-11)<br>– evidence that supports the presence or absence of each ASD diagnostic criterion<br>– evidence that supports the current severity level and specifiers (if DSM-5 criteria are utilized)<br>– the assessments conducted, including the name of the instrument, what it measures, the administering professional, the findings and their implications<br>– co-occurring conditions identified, diagnosed or requiring further investigation<br>– alternative conditions identified, diagnosed or requiring further investigation<br>– current developmental status / level of functioning across

	NICE	SIGN-HIS	NZ MOH	ACRC
	and written information about their right to:<br>a. short breaks and other respite care<br>b. formal career's assessment of their own physical and mental health needs, and how to access these.<br>- Offer families (including siblings) and careers an assessment of their own needs, including whether they have: personal, social and emotional support, practical support in their caring role, including short breaks and emergency plans, a plan for future care for the child or young person, including transition to adult services.<br>- When the needs of families and careers have been identified, discuss help available locally and, taking into account their preferences, offer information, advice,			multiple domains and potential level of functioning with supports<br>– activity-related and character strengths<br>– environmental facilitators and barriers<br>– highest priority support needs of the client and related goals<br>– suggested timeframe for the Comprehensive Needs Assessment to be repeated<br>– recommendations with sufficient details for the client to action:<br>– further assessments if required<br>– informal and formal supports required<br>– available funding and services.<br><br>[Consensus-based Recommendation, Grade 1]

	NICE	SIGN-HIS	NZ MOH	ACRC
	training and support, especially if they: need help with the personal, social or emotional care of the child or young person, including age-related needs such as self-care, relationships or sexuality, are involved in the delivery of an intervention for the child or young person in collaboration with health and social care professionals.			
**Interventions**				
**Problem minimization and avoidance**	- Do not use neurofeedback to manage speech and language problems in autistic children and young people.<br>- Do not use auditory integration training to manage speech and language problems in autistic children and young people.<br>- Do not use omega-3 fatty acids to manage sleep	Not mentioned	- Treatment should encourage functional development, teach skills for independent living, and minimize stress for the person with ASD, and their family and whānau.<br>- There is insufficient evidence to make any recommendation with respect to the use of the following biological	Not mentioned

	NICE	SIGN-HIS	NZ MOH	ACRC
	problems in autistic children and young people.<br>- Do not use the following interventions to manage autism in any context in children and young people: • secretin • chelation • hyperbaric oxygen therapy		agents, nutritional or other<br>approaches for ASD-specific symptoms in children with ASD.<br>- The opinion<br>of the Guideline Development Team is that these agents are unlikely to be useful:<br>combined vitamin B6-Mg<br>dimethylglycine<br>gluten and casein free (GCF) diet<br>omega-3/long chain polyunsaturated fatty acids<br>auditory integration training<br>holding therapy<br>options therapy<br>sensory integration therapies<br>Irlen lenses	

	NICE	SIGN-HIS	NZ MOH	ACRC
			- The use of Facilitated Communication for ASD-specific symptoms in children with ASD is not recommended.	
**Physical wellbeing and related needs**	- Ensure that all autistic children and young people have full access to health and social care services, including mental health services, regardless of their intellectual ability or any coexisting diagnosis.<br>- Health and social care professionals working with autistic children and young people in any setting should receive training in autism awareness and skills in managing autism, which should include:<br>— the nature and course of autism<br>— the nature and course of behavior that challenges in	- Healthcare professionals should be aware that factors in the social and physical environment may contribute to positive behaviors or those that challenge.	- Individualized support should be available to people with ASD who require assistance to manage their physical wellbeing and health care.<br>- Medical assessments should be comprehensive.<br>- A health-assessment profile for people with ASD should be developed and medical and health care practitioners trained in its use accordingly.<br>- The health-assessment profile should include:<br>screening for mental health issues and the careful surveillance for	- It is recommended that the process for assessing ASD concerns follow a holistic framework, where an individual is evaluated within their personal, activity and environmental contexts (as outlined, for example, by the World Health Organization's International Classification of Functioning, Disability and Health), and that referrals for further supports are based on an individual's functioning and needs, rather than their clinical diagnosis.

	NICE	SIGN-HIS	NZ MOH	ACRC
	autistic children and young people<br>— recognition of common coexisting conditions including:<br>◇ mental health problems such as anxiety and depression<br>◇ physical health problems such as epilepsy<br>◇ sleep problems<br>◇ other neurodevelopmental conditions such as attention deficit hyperactivity disorder (ADHD)<br>— the importance of key transition points, such as changing schools or health or social care services<br>— the child or young person's experience of autism and its impact on them<br>— the impact of autism on the family (including siblings) or carers		emergence of epilepsy<br>age-related prompts for screening for hearing loss, eyesight changes/ glaucoma, hypertension and metabolic syndrome<br>dietary and exercise guidelines to prevent secondary health issues, especially for those on medication<br>screening for motor, sensory and perceptual difficulties.<br>- People with ASD should have regular health checks, especially if they have<br>an intellectual disability or have ASD symptoms that may impair their ability to self-monitor or report potential health problems.	

	NICE	SIGN-HIS	NZ MOH	ACRC
	— the impact of the social and physical environment on the child or young person<br>— how to assess risk (including self-harm, harm to others, self-neglect, breakdown of family or residential support, exploitation or abuse by others) and develop a risk management plan<br>— the changing needs that arise with puberty (including the child or young person's understanding of intimate relationships and related problems that may occur, for example, misunderstanding the behaviour of others)<br>— how to provide individualized care and support and ensure a consistent approach is used across all settings		- Medical and health care practitioners should take into account the symptomatology of their ASD clients/patients, and adapt their practices and procedures accordingly.<br>- The dental needs of people with ASD in New Zealand should be investigated.<br>- People with ASD should be provided with factual information on dental hygiene tailored to their cognitive level.<br>Dentists should alter their processes and procedures to take into account the symptomatology of their ASD patients.<br>- The quality and quantity of sleep of people with ASD should be considered	

	NICE	SIGN-HIS	NZ MOH	ACRC
	— skills for communicating with an autistic child or young person.<br>- Take into account the physical environment in which autistic children and young people are<br>supported and cared for. Minimize any negative impact by:<br>— providing visual supports, for example, words, pictures or symbols that are meaningful for<br>the child or young person<br>— making reasonable adjustments or adaptations to the amount of personal space given<br>— considering individual sensory sensitivities to lighting, noise levels and the color of walls and furnishings.<br>• Make adjustments or adaptations to the processes of health or social care, for		by health care professionals, and be addressed therapeutically.<br>- Medication and behavioural treatment of sleep disorders should be considered.<br>- The effectiveness of medication and behavioural treatment of sleep disorders should be further investigated.<br>- Research should be undertaken to identify the needs of people with ASD<br>with regard to constipation, allergies, medication reactions, menstruation<br>and exercise.	

	NICE	SIGN-HIS	NZ MOH	ACRC
	example, arranging appointments at the beginning or end of the day to minimize waiting time, or providing single rooms for children and young people who may need a general anesthetic in hospital (for example, for dental treatment).			
**Non-pharmacological**				
**Educational assistance Placement Support**	- The intervention should be delivered by a trained professional. For pre-school children consider parent, career or teacher mediation. For school-aged children consider peer mediation.	- Education and skills interventions for parents of preschool children with ASD should be offered.	- Treatment plans should be comprehensive, and include behavioral needs, educational interventions, psychosocial treatments, communication, environmental and systems issues and the suitability (or not) of medication.<br>The student should be supported through the multiple transitions of	Not mentioned

	NICE	SIGN-HIS	NZ MOH	ACRC
			secondary school using schedules and other supports.<br>- A careful assessment of the skills which the young person requires for the transition to adult life in the community, leisure activities and the workplace should be undertaken. The outcome should inform the curriculum for the last few years of school.	
**Developmental interventions**	- Offer autistic children and young people support in developing coping strategies and accessing community services, including developing skills to access public transport, employment and leisure facilities.	- Access to support from staff trained in applied behavior analysis-based technologies (e.g. Picture Exchange Communication System, discrete trial training, task analysis, prompting, fading or shaping) to build independence in adaptive, communication and social	- Interventions and strategies based on applied behavior analysis (ABA) principles should be considered for all children with ASD.	Not mentioned

	NICE	SIGN-HIS	NZ MOH	ACRC
		skills should be considered for children with ASD.		
**Communication interventions**	- Consider a specific social-communication intervention for the core features of autism in children and young people that includes play-based strategies with parents, careers and teachers to increase joint attention, engagement and reciprocal communication in the child or young person.<br>- Strategies should:<br>a. be adjusted to the child or young person's developmental level<br>b. aim to increase the parents', careers', teachers' or peers' understanding of, and sensitivity and responsiveness to, the child or young person's patterns of communication and interaction	- Interventions to support communicative understanding and expression in individuals with ASD, such as the Picture Exchange Communication System and the use of environmental visual supports (e.g. in the form of pictures or objects), should be considered.<br>- Interventions to support social communication should be considered for children and young people with ASD, with the most appropriate intervention being assessed on an individual basis.<br>- Choice of interventions to support communication in children and young people with ASD should be informed by effective assessment.<br>- Adapting the communicative, social and physical	- Early intensive behavioral intervention (EIBI) should be considered as a treatment of value for young children with ASD to improve outcomes such as cognitive ability, language skills, and adaptive behavior.	Not mentioned

	NICE	SIGN-HIS	NZ MOH	ACRC
	c. include techniques of therapist modelling and video-interaction feedback<br>d. include techniques to expand the child or young person's communication, interactive play and social routines.	environments of children and young people with ASD may be of benefit (options include providing visual prompts, reducing requirements for complex social interactions, using routine, timetabling and prompting and minimizing sensory irritations).		
**Occupational therapy interventions**	Not mentioned	- Children and young people affected by ASD may benefit from occupational therapy, advice and support in adapting environments, activities and routines in daily life.	- Sensory issues in people with ASD should be identified and appropriately assessed by occupational therapists with experience in ASD. - - These assessments should lead to specific recommendations	Not mentioned
**Psycho-education for the family**	- Provide autistic children and young people, and their families and carers, with information about autism and its management and the support available on an	Not mentioned	- The values, knowledge, preferences and cultural perspectives of the family/<br>whānau should be respected and evident in services and resources.	- It is recommended that the process of assessing ASD concerns follow a strengths-focused approach, in which identifying the strengths, skills, interests, resources and support systems of the individual and their

	NICE	SIGN-HIS	NZ MOH	ACRC
	ongoing basis, suitable for the child or young person's needs and developmental level. This may include:<br>• contact details for local and national organizations that can provide:<br>— support and an opportunity to meet other people, including families or carers, with experience of autism<br>— information on courses about autism<br>— advice on welfare benefits, rights and entitlements<br>— information about educational and social support and leisure activities<br>• information about services and treatments available<br>• information to help prepare for the future, for example, transition to adult services.<br>- Make arrangements to support autistic children and young people and their family and carers during times of		- The stress experienced by the families and whānau of children with ASD should be acknowledged.<br>- The value of parent-led support networks should be recognized in helping parents to deal with the issues that they are facing following diagnosis and in supporting access to information.<br>- Teachers and other professionals should collect and appreciate the unique information about the child, which is held by the parent. This information should be incorporated into the planning of the child's education programme.	caregiver(s) and/or support people is recognized as being as important as identifying limitations

	NICE	SIGN-HIS	NZ MOH	ACRC
	increased need, including major life changes such as puberty, starting or changing schools, or the birth of a sibling.<br>- Explore with autistic children and young people, and their families and carers, whether they want to be involved in shared decision-making and continue to explore these issues at regular intervals. If children and young people express interest, offer a collaborative approach to treatment and care that takes their preferences into account.<br>- Offer all families (including siblings) and carers verbal and written information about their right to:<br>• short breaks and other respite care<br>• a formal carer's assessment of their own physical and mental health needs, and how to access these.		- The parents' role in interventions should be respectfully negotiated.<br>- Planning and evaluation of interventions should always take into account both family/whānau and child variables and outcomes.<br>- ASD-related counselling and/or advocacy services and education should be available to all family members and carers.<br>- Further research is needed to identify the needs of children parented by people with ASD and the needs of parents with ASD.	

	NICE	SIGN-HIS	NZ MOH	ACRC
	- Offer families (including siblings) and carers an assessment of their own needs,<br>including whether they have:<br>• personal, social and emotional support<br>• practical support in their caring role, including short breaks and emergency plans<br>• a plan for future care for the child or young person, including transition to adult services.<br>- When the needs of families and carers have been identified, discuss help available locally and, taking into account their preferences, offer information, advice, training and support, especially if they:<br>• need help with the personal, social or emotional care of the child or young person, including age-related needs			

	NICE	SIGN-HIS	NZ MOH	ACRC
	such as self-care, relationships or sexuality<br>• are involved in the delivery of an intervention for the child or young person in collaboration with health and social care professionals.			
**Special cases or comorbidities**	Not mentioned	Not mentioned	- When severe behaviors are evident, people with ASD need to be assessed for co-morbid conditions such as seizures, attention deficit hyperactivity disorder (ADHD), anxiety disorders, depression, and gastrointestinal problems.<br>- In severe or life-threatening situations, medication may be the best therapy.	Not mentioned
**Parent mediated interventions**	Not mentioned	- Parent-mediated intervention programs should be considered for children and young people of all ages who	Not mentioned	Not mentioned

	NICE	SIGN-HIS	NZ MOH	ACRC
		are affected by ASD, as they may help families interact with their child, promote development and increase parental satisfaction, empowerment and mental health.		
**Social communication and interaction intervention**	Not mentioned	- Interventions to support social communication should be considered for children and young people with ASD, with the most appropriate intervention being assessed on an individual basis.<br>- Adapting the communicative, social and physical environments of children and young people with ASD may be of benefit (options include providing visual prompts, reducing requirements for complex social interactions, using routine, timetabling and prompting and minimizing sensory irritations).	- Facilitated and structured social skills groups should be considered for high functioning children and young people with ASD.	Not mentioned

	NICE	SIGN-HIS	NZ MOH	ACRC
**Behavioral interventions**	- Assess factors that may increase the risk of behavior that challenges in routine assessment and care planning in autistic children and young people, including:<br>a. impairments in communication that may result in difficulty understanding situations or in expressing needs and wishes<br>b. coexisting physical disorders, such as pain or gastrointestinal disorders<br>c. coexisting mental health problems such as anxiety or depression and other neurodevelopmental conditions such as ADHD<br>d. the physical environment, such as lighting and noise levels<br>e. the social environment, including home, school and leisure activities<br>f. changes to routines or personal circumstances •	- Access to support from staff trained in applied behavior analysis-based technologies (e.g. Picture Exchange Communication System, discrete trial training, task analysis, prompting, fading or shaping) to build independence in adaptive, communication and social skills should be considered for children with ASD.	- Behavior management techniques should be used to intervene with problem behaviors following functional behavior assessment<br>- All behavioral interventions should be of good quality and incorporate the following principles: person-centered planning, functional assessment, positive intervention strategies, multifaceted interventions, focus on environment, meaningful outcomes, focus on ecological validity and systems-level intervention.<br>- Interventions and strategies based on applied behavior analysis (ABA)	Not mentioned

	NICE	SIGN-HIS	NZ MOH	ACRC
	developmental change, including puberty<br>g. exploitation or abuse by others<br>h. inadvertent reinforcement of behavior that challenges i. the absence of predictability and structure.		principles should be considered for all children with ASD.<br>- Early intensive behavioral intervention (EIBI) should be considered as a treatment of value for young children with ASD to improve outcomes such<br>as cognitive ability, language skills, and adaptive behavior.<br>- The feasibility of establishing publicly funded, ASD-specific behavioral<br>services should be investigated.	
**Cognitive Behavioral Therapy (CBT)**	- Consider the following for autistic children and young people with anxiety who have the verbal and cognitive ability to engage in a cognitive behavioral therapy (CBT) intervention:	- Cognitive behavioral therapy may be considered, using a group format where available and appropriate, to treat anxiety in children and young people with ASD and who have average verbal	- Cognitive behavior therapy should be considered as a suitable treatment for many behavioral, emotional and mental health difficulties.	Not mentioned

	NICE	SIGN-HIS	NZ MOH	ACRC
	a. group CBT adjusted to the needs of autistic children and young people<br>b. individual CBT for children and young people who find group-based activities difficult<br>- Consider adapting the method of delivery of CBT for autistic children and young people with anxiety to include:<br>a. emotion recognition training<br>b. greater use of written and visual information and structured worksheets<br>c. a more cognitively concrete and structured approach<br>d. simplified cognitive activities, for example, multiple-choice worksheets<br>e. involving a parent or carer to support the implementation of the intervention, for example, involving them in therapy sessions<br>f. maintaining attention by offering regular breaks g. incorporating the child or	and cognitive ability.<br>- The delivery of cognitive behavioral therapy should be adapted for people with ASD.<br>- Cognitive behavioral therapy can be considered as a means of treating a coexisting condition if recommended in guidelines for that condition.	- Cognitive behavior therapists should adapt their techniques to take into account the characteristics of people with ASD.	

	NICE	SIGN-HIS	NZ MOH	ACRC
	young person's special interests into therapy if possible.			
**Self-management**	Not mentioned	Not mentioned	Approaches should emphasise pivotal skills such as spontaneity, initiation, motivation and self-management.	Not mentioned
**Occupational Therapy**<br>**Parent training and coaching**	Not mentioned	- Children and young people affected by ASD may benefit from occupational therapy, advice and support in adapting environments, activities and routines in daily life.	Not mentioned	Not mentioned
**Sleep management**	- If an autistic child or young person develops a sleep problem offer an assessment that identifies:<br>a. what the sleep problem is (for example, delay in falling asleep, frequent waking, unusual behaviors, breathing problems or sleepiness during the day)	- Behavioral therapy should be considered for children and young people with ASD who experience sleep problems.<br>- Children with ASD who present with signs of possible obstructive sleep apnea, or sleep disordered breathing (loud snoring, choking or periodic stopping of breathing during sleep) should be	- The quality and quantity of sleep of people with ASD should be considered by health care professionals, and be addressed therapeutically.<br>- Medication and behavioural treatment of sleep disorders should be considered.	Not mentioned

	NICE	SIGN-HIS	NZ MOH	ACRC
	b. day and night sleep patterns, and any change to those patterns<br>c. whether bedtime is regular<br>d. what the sleep environment is like, for example: the level of background noise, use of a blackout blind, a television or computer in the bedroom, whether the child shares the room with someone, presence of comorbidities especially those that feature hyperactivity or other behavioral problems, levels of activity and exercise during the day, possible physical illness or discomfort (for example, reflux, ear or toothache, constipation or eczema), effects of any medication, any other individual factors thought to enhance or disturb sleep, such as emotional relationships or problems at school, and the impact of sleep and behavioral	referred to sleep medicine services for assessment.	- The effectiveness of medication and behavioural treatment of sleep disorders should be further investigated.<br>- Behavioural strategies (eg, sleep hygiene) should always be used in conjunction with melatonin..	

	NICE	SIGN-HIS	NZ MOH	ACRC
	problems on parents or carers and other family members.<br>- If the autistic child or young person snores loudly, chokes or appears to stop breathing while sleeping, refer to a specialist to check for obstructive sleep apnea.<br>- Develop a sleep plan (this will often be a specific sleep behavioral intervention) with the parents or carers to help address the identified sleep problems and to establish a regular night-time sleep pattern.<br>- Ask the parents or carers to record the child or young person's sleep and wakefulness throughout the day and night over a 2-week period.<br>- Use this information to modify the sleep plan if necessary and review the plan regularly until a regular sleep pattern is established.			

	NICE	SIGN-HIS	NZ MOH	ACRC
	- Do not use a pharmacological intervention to aid sleep unless: sleep problems persist despite following the sleep plan, sleep problems are having a negative impact on the child or young person and their family or carers.<br>-If the sleep problems continue to impact on the child or young person or their parents or carers, consider: referral to a pediatric sleep specialist and, short breaks and other respite care for one night or more.<br>- Short breaks may need to be repeated regularly to ensure that parents or carers are adequately supported. Agree the frequency of breaks with them and record this in the care plan.			
**Additional interventions**	Not mentioned	- Behavioral interventions may be considered to address a wide range of specific	Not mentioned	Not mentioned

	NICE	SIGN-HIS	NZ MOH	ACRC
		behaviors, including those that challenge, in children and young people with ASD, both to reduce symptom frequency and severity and to increase the development of adaptive skills.<br>- Healthcare professionals should be aware that some behaviors that challenge may be due to an underlying lack of skills development in the child/young person and also may represent an individual's strategy for coping with their difficulties and circumstances severity and to increase the development of adaptive skills.<br>- Healthcare professionals should be aware that factors in the social and physical environment may contribute to positive behaviors or those that challenge.		
**Feeding and Nutritional interventions**	- Be aware that feeding problems, including restricted diets can result in nutritional	- Gastrointestinal symptoms in children and young people with ASD should be managed	Not mentioned	Not mentioned

	NICE	SIGN-HIS	NZ MOH	ACRC
	deficiencies that may have serious consequences.<br>- Assess for any feeding, growth or nutritional problems, including restricted diets. Monitor and refer if needed.<br>- As part of a full nutritional assessment and monitoring, blood tests to check for nutritional deficiencies may be required.	in the same way as in children and young people without ASD.<br>- Advice on diet and food intake should be sought from a dietician for children and young people with ASD who display significant food selectivity and dysfunctional feeding behavior, or who are on restricted diets that may be adversely impacting on growth, or producing physical symptoms of recognized nutritional deficiencies or intolerances.		
**Support with challenging behavior**	- Develop a care plan with the child or young person and their families or careers that outlines the steps needed to address the factors that may provoke behavior that challenges, including:<br>a. treatment, for example, for coexisting physical, mental health and behavioral problems	Not mentioned	- Interventions should start early, as soon as challenging behaviors are of concern, and be proactive.<br>- The child or young person's programme should be individualized and designed to engage the child or young person and provide a highly	Not mentioned

	NICE	SIGN-HIS	NZ MOH	ACRC
	b. support, for example, for families or careers<br>c. necessary adjustments, for example, by increasing structure and minimizing unpredictability.<br>- If a child or young person's behavior becomes challenging, reassess factors identified in the care plan and assess for any new factors that could provoke the behavior.<br>- Offer the following to address factors that may trigger or maintain behavior that challenges:<br>a. treatment for physical disorders, or coexisting mental health and behavioral problems<br>b. interventions aimed at changing the environment, such as: providing advice to families and careers, making adjustments or adaptations to the physical surroundings		supportive environment.<br>- Educational interventions should incorporate principles of positive behavior support, particularly a focus on understanding the function of the child's behavior.<br>- Physically aversive procedures should not be used.<br>- All school staff should understand the goals of a child or young person's behavior-support plan.	

	NICE	SIGN-HIS	NZ MOH	ACRC
	- If behavior remains challenging despite attempts to address the underlying possible causes, consult senior colleagues and undertake a multidisciplinary review.<br>- At the multidisciplinary review, take into account the following when choosing an intervention for behavior that challenges:<br>a. the nature, severity and impact of the behavior<br>b. the child or young person's physical and communication needs and capabilities<br>c. the environment<br>d. the support and training that families, careers or staff may need to implement the intervention effectively<br>e. the preferences of the child or young person and the family or careers<br>f. the child or young person's experience of, and response to, previous interventions.			

	NICE	SIGN-HIS	NZ MOH	ACRC
	- If no coexisting mental health or behavioral problem, physical disorder or environmental problem has been identified as triggering or maintaining the behavior that challenges, offer the child or young person a psychosocial intervention (informed by a functional assessment of behavior) as a first-line treatment.<br>- The functional assessment should identify:<br>a. factors that appear to trigger the behavior<br>b. patterns of behavior<br>c. the needs that the child or young person is attempting to meet by performing the behavior<br>d. the consequences of the behavior (that is, the reinforcement received as a result of the behavior).			

	NICE	SIGN-HIS	NZ MOH	ACRC
	- Psychosocial interventions for behavior that challenges should include:<br>a. clearly identified target behavior<br>b. a focus on outcomes that are linked to quality of life<br>c. assessment and modification of environmental factors that may contribute to initiating or maintaining the behavior<br>d. a clearly defined intervention strategy that takes into account the developmental level and coexisting problems of the child or young person<br>e. a specified timescale to meet intervention goals (to promote modification of intervention strategies that do not lead to change within a specified time)<br>f. a systematic measure of the target behavior taken before and after the intervention to			

	NICE	SIGN-HIS	NZ MOH	ACRC
	ascertain whether the agreed outcomes are being met<br>g. consistent application in all areas of the child or young person's environment<br>h. agreement among parents, careers and professionals in all settings about how to implement the intervention.			
**Pharmacological Interventions**				
**General principles**	- Do not use the following interventions for the management of core features of autism in children and young people: antipsychotics, antidepressants, anticonvulsants, exclusion diets (such as gluten- or casein-free diets).	Not mentioned	Not mentioned	Not mentioned
**Antipsychotics (conventional and atypical)**	- Consider antipsychotic medication for managing behavior that challenges in autistic children and young people when psychosocial or other interventions are insufficient or could not be	- Antipsychotics (including second-generation antipsychotics) should not be used to manage the core symptoms of ASD in children and young people.<br>- Second-generation antipsychotics may be	- The antipsychotic medication risperidone is effective in reducing aggressive behavior, irritability and self-injurious behavior in children with ASD.	Not mentioned

	NICE	SIGN-HIS	NZ MOH	ACRC
	delivered because of the severity of the behavior.<br>- Antipsychotic medication should be initially prescribed and monitored by a pediatrician or psychiatrist who should:<br>a. identify the target behavior<br>b. decide on an appropriate measure to monitor effectiveness, including frequency and severity of the behavior and a measure of global impact<br>c. review the effectiveness and any side effects of the medication after 3–4 weeks<br>d. stop treatment if there is no indication of a clinically important response at 6 weeks.<br>- If antipsychotic medication is prescribed: start with a low dose, use the minimum effective dose needed, and regularly review the benefits of the antipsychotic	considered to reduce irritability and hyperactivity in children and young people with ASD in the short term (eight weeks).<br>- Patients and their careers should be advised of potential side effects before treatment is started.<br>- Children prescribed second-generation antipsychotics should be reviewed after three or four weeks of medication.<br>- If there is no clinically important response at six weeks treatment should be stopped.	- It should be used with caution because of the high risk of adverse effects and the uncertainty about long-term effects.<br>- Monitoring for side effects should be carried out on a regular basis.	

	NICE	SIGN-HIS	NZ MOH	ACRC
	medication and any adverse events.<br>- When choosing antipsychotic medication, take into account side effects, acquisition costs, the child or young person's preference (or that of their parent or career where appropriate) and response to previous treatment with an antipsychotic.<br>- When prescribing is transferred to primary or community care, the specialist should give clear guidance to the practitioner who will be responsible for continued prescribing about: the selection of target behaviors, monitoring of beneficial and side effects, the potential for minimally effective dosing, the proposed duration of treatment, and plans for stopping treatment.			
**Stimulants**		- Methylphenidate may be considered for management of	Not mentioned	Not mentioned

	NICE	SIGN-HIS	NZ MOH	ACRC
		attention difficulties/hyperactivity in children or young people with ASD.<br>- Use of a test dose to assess if methylphenidate is tolerated could be considered in children prior to any longer trial.<br>- Side effects should be carefully monitored		
**Antidepressants-Selective Serotonin Reuptake Inhibitors (SSRIs)**	Not mentioned	- Selective serotonin reuptake inhibitors should not be used to manage core features of ASD (e.g. repetitive behaviors) in children and young people.<br>- Selective serotonin reuptake inhibitors should be considered for children and young people with comorbid symptoms on a case-by-case basis.	- SSRIs (e.g., fluoxetine) may be effective for some children with ASD and high anxiety and/or obsessive symptoms. However, without quality evidence, these drugs should be used with caution and careful monitoring.<br>- There is insufficient evidence to make any recommendation about the use of other types of antidepressants.	Not mentioned

	NICE	SIGN-HIS	NZ MOH	ACRC
**Anticonvulsants and mood stabilizers**	Not mentioned	Not mentioned	Not mentioned	Not mentioned
**Alpha 2 agonists, Antianxiety and benzodiazepines**		Not mentioned	There is insufficient evidence to make any recommendation with respect to the use of the following drugs for ASD-specific symptoms in children with ASD. However, these medications are in current use, and may be used by experienced clinicians, who maintain up to date knowledge of the literature:<br>clonidine<br>sedatives (benzodiazepines, antihistamines).	Not mentioned
**Sleep medication**	- If a pharmacological intervention is needed to aid sleep, consider melatonin and: only use it following consultation with a specialist	- In children with ASD who have sleep difficulties which have not resolved following behavioral interventions, a trial	- Melatonin can be recommended for use in children and young people with ASD who are	Not mentioned

	NICE	SIGN-HIS	NZ MOH	ACRC
	pediatrician or psychiatrist with expertise in the management of autism or paediatric sleep medicine, use it in conjunction with non-pharmacological interventions, and regularly review to evaluate the ongoing need for a pharmacological intervention and to ensure that the benefits continue to outweigh the side effects and risks.	of melatonin to improve sleep onset should be considered.<br>- Use of melatonin should follow consultation with a pediatrician or psychiatrist with expertise in the management of sleep medicine in children and/or ASD, and be in conjunction with behavioral interventions.<br>- Melatonin prescription should be reviewed regularly in the context of any emerging possible side effects and/or reduced therapeutic effect.<br>- Children with ASD who present with signs of possible obstructive sleep apnea, or sleep-disordered breathing (loud snoring, choking or periodic stopping of breathing during sleep) should be referred to sleep medicine services for assessment.	experiencing significant sleep problems.<br>- Benefits and adverse effects of longer term treatment of melatonin require further investigation	

	NICE	SIGN-HIS	NZ MOH	ACRC
		- Obtain an adequate baseline sleep diary before any trial of melatonin.<br>- Continue sleep hygiene measures (bedtime and wake-up routine, avoidance of day-time sleep) and a sleep diary, during any medication trial.		
**Crisis management**	Not mentioned	Not mentioned	Not mentioned	Not mentioned
**Mental health and forensic services**	Not mentioned	Not mentioned	Adults with ASD who are patients of adult mental health or forensic services should be supported to overcome fears and given information on their rights and advocacy services.	Not mentioned
**Transition of care**	- Local autism teams should ensure that autistic young people who are receiving treatment and care from child and adolescent mental health services (CAMHS) or child health services are reassessed at around 14 years to establish	Not mentioned	Not mentioned	Not mentioned

	NICE	SIGN-HIS	NZ MOH	ACRC
	the need for continuing treatment into adulthood.<br>- If continuing treatment is necessary, make arrangements for a smooth transition to adult services and give information to the young person about the treatment and services they may need.<br>- The timing of transition may vary locally and individually but should usually be completed by the time the young person is 18 years. Variations should be agreed by both child and adult services.<br>- As part of the preparation for the transition to adult services, health and social care professionals should carry out a comprehensive assessment of the autistic young person.<br>- The assessment should make best use of existing documentation about personal, educational, occupational, social and			

	NICE	SIGN-HIS	NZ MOH	ACRC
	communication functioning, and should include assessment of any coexisting conditions, especially depression, anxiety, ADHD, obsessive-compulsive disorder (OCD) and global delay or intellectual disability in line with the NICE guideline on autism in adults.<br>- For young people aged 16 or older whose needs are complex or severe, use the care programme approach (CPA) in England, or care and treatment plans in Wales, as an aid to transfer between services.<br>- Involve the young person in the planning and, where appropriate, their parents or carers.<br>- Provide information about adult services to the young person, and their parents or carers, including their right to a social care assessment at age			

	NICE	SIGN-HIS	NZ MOH	ACRC
	- During transition to adult services, consider a formal meeting involving health and social care and other relevant professionals from child and adult services.			
**Recommendations for future research and health policy makers**		Not mentioned		Not mentioned
**Living in the community**	- Offer autistic children and young people support in developing coping strategies and accessing community services, including developing skills to access public transport, employment and leisure facilities.	Not mentioned	- Careful and timely attention should be paid to planning for people with ASD leaving school and moving into further and post-compulsory education, work (paid or unpaid) or vocational services.<br>- Providers of further and post-compulsory education should ensure that their members of staff are aware of the specific educational	Not mentioned

	NICE	SIGN-HIS	NZ MOH	ACRC
			needs of people with ASD.<br>- Work (paid and unpaid) should be considered an option for all people with ASD, regardless of their intellectual ability.<br>- Any known support needs of people with ASD, including those relating to cognitive ability, should be taken into account when transitioning into any work environment.<br>- Supported employment services for people with ASD should be developed.<br>- Vocational services of a high standard should be available to people with ASD who are not ready or able to access post-compulsory education and work.	

	NICE	SIGN-HIS	NZ MOH	ACRC
**Recreation and leisure**	Not mentioned	Not mentioned	- All children and adults with ASD should have access to leisure facilities and meaningful activity tailored to their needs and interests: this is supported by person-centered plans designed by staff who have received specialist education for the role using strategies to promote social inclusion.<br>- Plans should be regularly evaluated.<br>- Leisure and recreation planning should be included in a student/young person's transition programme and this information shared with postschool providers.	Not mentioned
**Contact with the justice system**		Not mentioned	- Young people and adults with ASD should be taught their legal	Not mentioned

	NICE	SIGN-HIS	NZ MOH	ACRC
			rights and be prepared in advance with information should they ever have contact with the police and legal authorities.<br>- Appropriate resources and training should be developed to help with this.<br>- People with ASD involved in disputes within the Family Court should seek support from solicitors and advocacy services with knowledge and experience in ASD.	

Supplementary Table S6. Classification of the strength of agreement among the four raters against the four CPGs

	Poor	Fair	Good	Very good	Excellent	Sum of scores	Sum of OA1 scores	Overall assessment 1 (OA1)
**SIGN-HIS**	1	0	12	7	4	575	26	Good
**NICE**	0	0	5	15	4	591	27	Very Good
**NZ MOH**	2	0	9	12	1	529	25	Very Good
**Autism CRC**	1	0	10	8	5	594	26	Good
